# Identification of a serum proteomic biomarker panel using diagnosis specific ensemble learning and symptoms for early pancreatic cancer detection

**DOI:** 10.1371/journal.pcbi.1012408

**Published:** 2024-08-29

**Authors:** Alexander Ney, Nuno R. Nené, Eva Sedlak, Pilar Acedo, Oleg Blyuss, Harry J. Whitwell, Eithne Costello, Aleksandra Gentry-Maharaj, Norman R. Williams, Usha Menon, Giuseppe K. Fusai, Alexey Zaikin, Stephen P. Pereira

**Affiliations:** 1 Institute for Liver and Digestive Health, University College London, London, United Kingdom; 2 Department of Women’s Cancer, EGA Institute for Women’s Health, University College London, London, United Kingdom; 3 Cancer Institute, University College London, London, United Kingdom; 4 Department of Statistical Science, University College London, London, United Kingdom; 5 Center for Cancer Prevention, Detection and Early Diagnosis, Wolfson Institute of Population Health, Queen Mary University of London, London, United Kingdom; 6 Department of Pediatrics and Pediatric Infectious Diseases, Institute of Child´s Health, Sechenov First Moscow State Medical University (Sechenov University), Moscow, Russia; 7 National Phenome Centre and Imperial Clinical Phenotyping Centre, Department of Metabolism, Digestion and Reproduction, IRDB, Building Imperial College London, London, United Kingdom; 8 Section of Bioanalytical Chemistry, Division of Systems Medicine, Department of Metabolism, Digestion and Reproduction, Sir Alexander Fleming Building, Imperial College London, London, United Kingdom; 9 Department of Molecular and Clinical Cancer Medicine, University of Liverpool, Liverpool, United Kingdom; 10 MRC Clinical Trials Unit at UCL, Institute of Clinical Trials and Methodology, University College London, London, United Kingdom; 11 Division of Surgery & Interventional Science, University College London, London, United Kingdom; 12 HPB & Liver Transplant Unit, Royal Free London, London, United Kingdom; 13 Institute for Cognitive Neuroscience, University Higher School of Economics, Moscow, Russia; 14 Department of Mathematics, University College London, London, United Kingdom; 15 Centre for Cognition and Decision making, Institute for Cognitive Neuroscience, HSE University, Moscow, Russia; 16 Life Improvement by Future Technologies (LIFT) Center, Skolkovo, Moscow, Russia; Universidad de Costa Rica, COSTA RICA

## Abstract

**Background:**

The grim (<10% 5-year) survival rates for pancreatic ductal adenocarcinoma (PDAC) are attributed to its complex intrinsic biology and most often late-stage detection. The overlap of symptoms with benign gastrointestinal conditions in early stage further complicates timely detection. The suboptimal diagnostic performance of carbohydrate antigen (CA) 19–9 and elevation in benign hyperbilirubinaemia undermine its reliability, leaving a notable absence of accurate diagnostic biomarkers. Using a selected patient cohort with benign pancreatic and biliary tract conditions we aimed to develop a data analysis protocol leading to a biomarker signature capable of distinguishing patients with non-specific yet concerning clinical presentations, from those with PDAC.

**Methods:**

539 patient serum samples collected under the Accelerated Diagnosis of neuro Endocrine and Pancreatic TumourS (ADEPTS) study (benign disease controls and PDACs) and the UK Collaborative Trial of Ovarian Cancer Screening (UKCTOCS, healthy controls) were screened using the Olink Oncology II panel, supplemented with five in-house markers. 16 specialized base-learner classifiers were stacked to select and enhance biomarker performances and robustness in blinded samples. Each base-learner was constructed through cross-validation and recursive feature elimination in a discovery set comprising approximately two thirds of the ADEPTS and UKCTOCS samples and contrasted specific diagnosis with PDAC.

**Results:**

The signature which was developed using diagnosis-specific ensemble learning demonstrated predictive capabilities outperforming CA19-9, the only biomarker currently accepted by the FDA and the National Comprehensive Cancer Network guidelines for pancreatic cancer, and other individual biomarkers and combinations in both discovery and held-out validation sets. An AUC of 0.98 (95% CI 0.98–0.99) and sensitivity of 0.99 (95% CI 0.98–1) at 90% specificity was achieved with the ensemble method, which was significantly larger than the AUC of 0.79 (95% CI 0.66–0.91) and sensitivity 0.67 (95% CI 0.50–0.83), also at 90% specificity, for CA19-9, in the discovery set (p = 0.0016 and p = 0.00050, respectively). During ensemble signature validation in the held-out set, an AUC of 0.95 (95% CI 0.91–0.99), sensitivity 0.86 (95% CI 0.68–1), was attained compared to an AUC of 0.80 (95% CI 0.66–0.93), sensitivity 0.65 (95% CI 0.48–0.56) at 90% specificity for CA19-9 alone (p = 0.0082 and p = 0.024, respectively). When validated only on the benign disease controls and PDACs collected from ADEPTS, the diagnostic-specific signature achieved an AUC of 0.96 (95% CI 0.92–0.99), sensitivity 0.82 (95% CI 0.64–0.95) at 90% specificity, which was still significantly higher than the performance for CA19-9 taken as a single predictor, AUC of 0.79 (95% CI 0.64–0.93) and sensitivity of 0.18 (95% CI 0.03–0.69) (p = 0.013 and p = 0.0055, respectively).

**Conclusion:**

Our ensemble modelling technique outperformed CA19-9, individual biomarkers and indices developed with prevailing algorithms in distinguishing patients with non-specific but concerning symptoms from those with PDAC, with implications for improving its early detection in individuals at risk.

## Author summary

Pancreatic ductal adenocarcinoma (PDAC) has one of the lowest 5-year survival rates among cancers, primarily due to its complex biology and late-stage diagnosis. Early symptoms often mimic benign gastrointestinal conditions, complicating timely detection. The standard biomarker, carbohydrate antigen (CA) 19–9, is not reliable due to its suboptimal performance and elevation in benign conditions, highlighting the need for better diagnostic tools. In our study, we aimed to develop a biomarker signature to distinguish between benign pancreatic/biliary conditions and PDAC using serum samples from the Accelerated Diagnosis of neuro Endocrine and Pancreatic Tumours and UK Collaborative Trial of Ovarian Cancer Screening studies.

We screened 539 patient serum samples with state-of-the-art biomarker panels and developed a robust predictive signature by applying specialized machine learning methods. This new signature significantly outperformed CA19-9 and other panels reported in the literature.

Our findings suggest that this new biomarker signature can improve early detection of PDAC in patients with ambiguous clinical symptoms, potentially leading to better outcomes for those at risk.

## Introduction

Pancreatic ductal adenocarcinoma (PDAC) ranks as the seventh primary cause of cancer-related mortality [1,2]. Projections suggest that by 2030, mortality rates from PDAC will exceed that of other prevalent cancers, a shift which is attributed to an increasing incidence of obesity, diabetes mellitus, alcohol consumption in some regions (Europe, North America, and Oceania), advancements in detection and institution of screening initiatives that facilitate the timely identification of more common cancers [1–3].

The overall 5-year survival for pancreatic cancer (PC) patients is less than 10%. These figures improve in patients diagnosed with pre-invasive lesions (intraepithelial neoplasia, mucinous cystic lesions) or small tumours (< 2cm) detected at a localised stage [4]. Patients with resectable disease are only identified in less than 20% of cases and advances in early detection strategies hold potential for improving these dismal figures [5,6]. The relatively low incidence and lifetime risk for PC in the general population (1.3%) preclude asymptomatic, average-risk adult (>50 age) screening, and efforts are rather focused on high-risk populations [6–8]. Internationally, screening and surveillance is therefore recommended only in high-risk individuals (genetically predisposed, family history and high-risk pancreatic cysts), where a lifetime risk of at least 5% justifies their surveillance [6,7,9,10]. While surveillance in these high-risk cohorts is consensus, we also reported on symptomatic cohorts in which the increased risk could justify investigations, as an additional risk group [6,11].

Existing evidence regarding the effect of timely diagnosis on outcomes in PDAC are limited, mostly due to the lack of randomisation, appropriate statistical considerations and homogenisations of study populations, and the topic remains an area of strong debate [12]. Yet, it is very likely that prompt identification of PC would improve its prognosis [12–14].

The reality of the situation however is that disease rarity, the presence of non-localising symptoms, the relatively low positive predictive values even for cancer specific ‘red-flag’ and advanced symptoms (e.g. weight loss, painless jaundice of 4–13%) challenge timely recognition in primary care settings, and a substantial number of PC patients are diagnosed following prolonged periods of clinical uncertainty [15,16]. Previous case-control primary care studies associated various abdominal symptoms and increased frequency of primary care consultations with PDAC, over the two years preceding its diagnosis [11,17,18]. These data suggest another potential window of opportunity for acceleration of PC detection.

In roughly 30% of patients, PC manifests in the form of jaundice indicating tumour induced biliary obstruction, which is more evident in pancreatic head tumours [19]. Together with significant weight loss, these frequently represent an already advanced disease. Although most often explained by benign aetiologies, symptoms such as back or epigastric pain, dyspepsia, anorexia, bloating, changes in consistency of stool, weight loss and anxiety/depression may also indicate an underlying pancreatic malignancy [11,17–20]. Such symptoms in adults (age > 60 years) with lifestyle factors (including heavy alcohol and tobacco consumption, obesity) and on the background of new or long-standing diabetes and chronic pancreatitis, are worrisome [6,11,18].

To accelerate and improve cancer detection rates in the UK, ‘electronic cancer decision support tools’ (eCDST) have been developed to support primary care clinicians in fast tracking investigations in cases of suspected cancer [21–23]. Risk prediction algorithms such as QCancer [21–23] combine symptoms data, patient risk factors and laboratory tests to predict a risk of undiagnosed cancers of various anatomical sites (colon, pancreas, renal, gastro-oesophageal and ovarian). These are digitally available for primary care physicians through patient record and data management portals [21–24] and where higher risk justifies further investigations, could be combined with blood biomarker panels for further risk stratification prior to more invasive workup.

When suspected, establishing a diagnosis will involve measurement of the serum marker CA19-9, cross-sectional (computed tomography or magnetic resonance) imaging and histopathology (endoscopic ultrasound guided tissue biopsy; EUS-FNB). CA19-9 is most reliable as a marker of tumour resectability, prognosis and monitoring of disease progression [25,26], but as a diagnostic marker it performs poorly (median sensitivity and specificity of ~80%; AUC = 0.82), particularly in stage I/II disease and in Lewis body negative patients [27,28]. The development of reliable and accurate diagnostic biomarkers is essential for risk stratification and prioritisation of further investigations, as well as justification of invasive interventions where the findings on imaging are unequivocal [29].

Recent research has explored blood-based diagnostic biomarkers including proteins, micro-RNAs, circulating tumor cells and DNA methylation patterns, yet remain unvalidated in clinically representative cohorts [30,31]. Their aberrant expression in both inflammatory and malignant processes further challenge their discriminative properties. Multi-cancer early detection tests like CancerSEEK [32] and Galleri are emerging [33,34]. These analyse circulating DNA for genetic mutations and proteins or methylation patterns associated with cancer. CancerSEEK has shown 67% sensitivity for 12 cancers at 99% specificity, with 72% sensitivity for pancreatic cancer (stages I–III) and 83.7% sensitivity for pancreatic ductal adenocarcinoma (PDAC) detection. However, sensitivity varies across cancer types and additional larger validation studies [34] are needed before considering them for widespread screening [35].

Using serum samples collected from a selected study cohort with benign pancreatic and biliary tract conditions and applying robust machine learning stacked modelling, we therefore developed a novel serum biomarker signature capable of differentiating PC patients from healthy individuals and patients with benign abdominal conditions presenting with non-specific yet concerning symptoms for pancreatic cancer, at higher rates than CA19-9 and other state-of-the-art biomarkers.

## Results

### Data set characteristics

**Fig 1:**
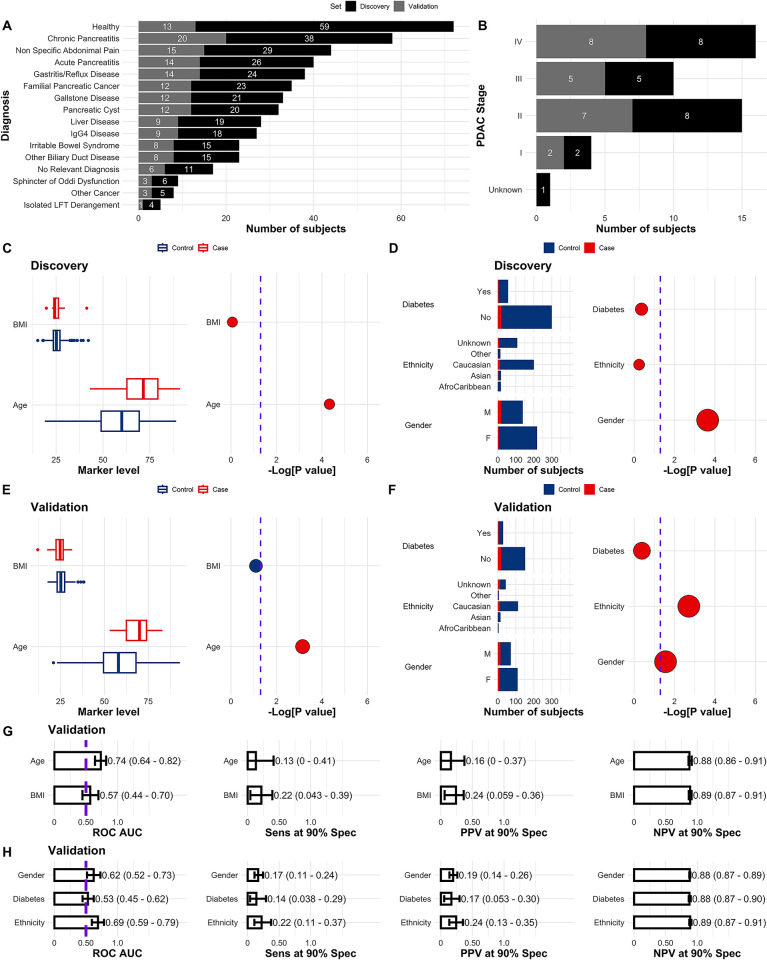
Characteristics of the discovery and validation sets. Number of controls across the discovery and validation sets **(A),** number of PDAC cases per stage **(B),** and association of BMI, Age, Diabetes, Ethnicity and Gender with PDAC status **(C-F).** In C, D, E and F dot sizes correspond to odds ratios and are colour coded according to their respective values, i.e., blue if OR<1 and red if OR>1. p values were calculated according to a logistic regression model with a bias reduction method. Purple dashed lines correspond to -Log[0.05]. **G** Receiver Operating Characteristic (ROC) Area Under the Curve (AUC), Sensitivity (Sens), Positive Predictive Value (PPV) and Negative Predictive Value (NPV) at 90% Specificity (Spec) performance of single marker models, i.e. BMI and Age, in the validation set. **H** Similar to A but for Gender, Ethnicity and Diabetes. Performances were calculated with the respective single feature models developed in the discovery set. The ROC AUC significance threshold is also represented by a purple dashed line at 0.5. Error bars in figures corresponding to the validation set are the 95% Confidence Intervals (CI), calculated by stratified bootstrapping 2000 times. See Statistical Analysis in Materials and methods for further details and Tables A, B and N in S1 Appendix.

In the full set of samples collected from the ADEPTS cohort, age at the time of sample collection, 57.44 (range from 19.00 to 93.00) for controls and 69.72 (range from 43.00 to 91.00) for PDAC cases, emerged as a risk factor (OR = 1.06 (95% CI 1.04–1.09), p = 2.47×10^−7^) (Table 1). As a predictor in a logistic regression model age achieved a ROC AUC of 0.73 (95% CI 0.66–0.79), with a cut-off at 61.5 years (calculated using the Youden’s J statistic). This finding was also observed in both the discovery (Fig 1 and Table A in S1 Appendix, ROC AUC 0.74 (95% CI 0.64–0.83), cut-off at 70) and validation sets (Fig 1 and Table B in S1 Appendix, 0.74 (95% CI 0.64–0.82), cut-off at 60), which incorporated not only ADEPTS samples but also healthy control samples collected from UKCTOCS [36]. In our past research which was focused exclusively on UKCTOCS longitudinal samples, age similarly emerged as a risk factor for PDAC [37]. Furthermore, gender (OR = 2.72 (95% CI 1.46–5.27), p = 0.0015) and ethnicity taken as a one-hot encoded variable (OR = 2.02 (95% CI 1.34–3.03), p = 6.56×10^−4^) were also confirmed as significantly associated with an increased risk of PDAC (Table 1). In the whole set of samples collected from the ADEPTS cohort, men had a 2.72-fold risk of PDAC compared to their female counterparts. Individuals of Caucasian ethnicity demonstrated a decreased risk of PDAC in a one versus rest calculation (OR = 0.38 (95% CI 0.20–0.69), p = 0.0018) and no significant association was found between PDAC risk and Asian or Afro-Caribbean ethnicity in the ADEPTS dataset under the same modelling framework (Table 1). The association of gender and PDAC was also confirmed in the discovery (OR = 4.98 (95% CI 2.08–13.50), p = 0.00023, Fig 1 and Table A in S1 Appendix) and validation sets (OR = 2.65 (95% CI 1.11–6.58), p = 0.028 Fig 1 and Table B in S1 Appendix), but ethnicity, taken as a one-hot encoded variable, remained a significant predictor of PDAC only in the validation set (OR = 2.66 (95% CI 1.42–5.17), p = 0.0020) (Fig 1 and Table B in S1 Appendix), which as was highlighted above also includes healthy control UKCTOCS samples. Within the group of the clinical covariates only age and gender are significant predictors of PDAC in both the discovery and validation set (Fig 1), with only age achieving a significant AUC in the validation set between these two. However, this was concomitant with remarkably low sensitivity (Sens), positive predictive (PPV) value and negative predictive value (NPV) at 90% specificity (Spec): AUC 0.74 (95% CI 0.64–0.82), Sens 0.13 (95% CI 0–0.39), PPV 0.16 (95% CI 0–0.36), NPV 0.88 (95% CI 0.86–0.91).

**Table 1:** Cohort characteristics. The data set used to develop and test the classifiers is a combination of samples collected from the ADEPTS cohort and selected controls from the UKCTOCS cohort. BMI: Body Mass Index. See Study Design in Materials and methods section for additional details. Odds ratio (OR) and respective 95% confidence intervals are also provided in the p value column.

Variable	Cases	Controls	*p* value
**ADEPTS**			
No. samples	46	421	-
Stage I	4	-	
Stage II	15	-	
Stage III	10	-	
Stage IV	16	-	
Unknown	1	-	
Mean age at sample draw (yr) (range)	69.72 (43.00–91.00)	57.44 (19.00–93.00)	2.47×10^−7^ OR = 1.06 (1.04–1.09)
Mean BMI (kg/m2) (range)	24.84 (12.04–41.35)	25.30 (15.22–39.45)	0.47 OR = 0.97 (0.88–1.06)
Gender			
Male	31	180	0.0015 OR = 2.72 (1.46–5.27)
Female	15	241	
Diabetes			
Yes	10 (8Type II, 1 Type I, 1 unspecified)	75 (34 Type II, 41 unspecified)	0.44 OR = 1.34 (0.62–2.70)
No	36	346	
Ethnicity			
Caucasian	21	291	6.56×10^−4^ OR = 2.02 (1.34–3.03)
Unknown	21	60	
Asian	3	30	
Other	2	18	
Afro/Caribbean	0	22	
**UKCTOCS**			
No. samples	-	72	-
Mean age at sample draw (yr) (range)	-	62.95 (50.44–76.86)	-
Mean BMI (kg/m2) (range)	-	26.53 (17.91–42.19)	-
Gender			
Male	-	-	-
Female	-	72	
Diabetes			
Yes	-	3 (3 Type II)	-
No	-	69	
Ethnicity			
Unknown	0	72	-

### Development of a PDAC biomarker signature in the presence of confounding conditions

To aid the early detection of this cancer in individuals at risk, we aimed to develop a biomarker signature that could be used to differentiate between suspected PDACs and benign biliary conditions that often overlap in clinical presentation. We applied a uniquely developed ensemble learning model, with a logistic regression stacking layer (see Fig A in S1 Appendix and statistical analysis in the Materials and methods section), to a set of 539 serum samples (493 controls and 46 PDAC cases) which were analysed using the Olink Oncology II panel as well as four additional biomarkers we previously reported on [37]. These included IL6ST, VWF, THBS2 and CA19-9. The oncogenic and prognostic glycolytic enzyme PKM2 was additionally selected based on our past report of its diagnostic utility in biliary tract cancer patients [38–40].

**Fig 2:**
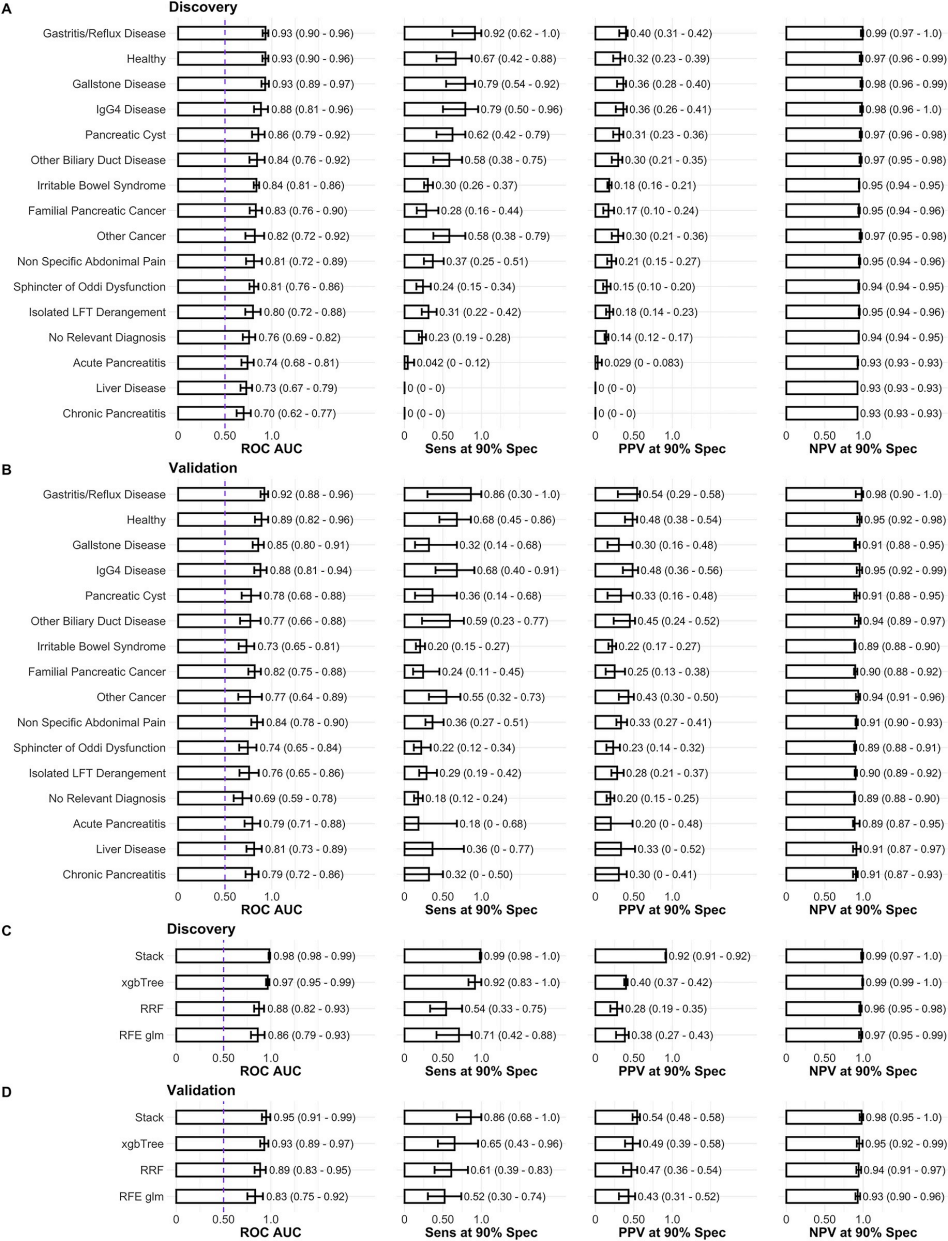
Performance of individual base-learner classifiers, stack ensemble and state-of-the art algorithms. **A** Base-learners performance in the discovery set. Each base-learner classifier was developed by training with a recursive feature elimination technique (RFE) and logistic regression (glm) in samples belonging to each specific diagnosis class against the same 24 PDACs in the discovery set. The performance reported in A is, nevertheless, of each classifier in the whole discovery set. The performances reported in **B** correspond to the base-learners developed in the discovery set but applied to the whole validation set. In **C** and **D** the performance of the ensemble stack based on the base-learners presented in A and B, as well as of state-of-the-art algorithms (xgbTree, RRF and RFE glm) is reported in the discovery and held-out validation sets, respectively. xgbTree, RRF and RFE glm were trained in the whole discovery set, which contrasts with the ensemble algorithm. * Receiver Operating Characteristic (ROC) Area Under the Curve (AUC), Sensitivity (Sens), Positive Predictive Value (PPV) and Negative Predictive Value (NPV) at 90% Specificity (Spec).*

The application of stacked ensemble modelling as presented herein bolsters the robustness of predictive outcomes, enhancing the performance of biomarker panels through the incorporation of serum biomarker levels and relevant clinical covariates for distinct diagnostic classes. Each base classifier within the ensemble is designed to provide a specialized distinction between confounding diagnoses and PDAC, thereby establishing a heterogeneous set of classifiers that facilitates the precise identification of PDAC (see statistical analysis section in Materials and methods). Previous studies have attested to the beneficial role of ensemble methods in augmenting early detection of PDAC against only healthy controls [37]. The implementation of stacked (Stack, Fig 2), specialized classifiers, developed within the discovery set, generated a biomarker signature capable of predicting PDAC with an AUC of 0.98 (95% CI 0.98–0.99), sensitivity of 0.99 (95% CI 0.98–1), PPV 0.92 (95% CI 0.91–0.92) and NPV 0.99 (95% CI 0.97–1) at 90% specificity. In contrast, the predictive efficacy of CA19-9 in the discovery set taken as a single predictor under a logistic regression model was 0.79 (95% CI 0.66–0.91) (p = 0.0016 under a one-sided bootstrap test comparing the two AUCs), sensitivity 0.67 (95% CI 0.50–0.83), PPV 0.32 (95% CI 0.26–0.38) and NPV of 0.97 (95% CI 0.96–0.99) at 90% specificity (see Table C in S1 Appendix). Amongst all biomarkers, CA19-9 demonstrated the most significant association (refer to Table C and Fig B in S1 Appendix for univariate trend associations across the discovery set), and one of the highest performances in the validation set (Fig B and Table D in S1 Appendix).

In the validation set, the ensemble signature predicted PDAC with an AUC of 0.95 (95% CI 0.91–0.99), sensitivity 0.86 (95% CI 0.68–1), PPV 0.54 (95% CI 0.48–0.58) and NPV of 0.98 (95% CI 0.95–1) at 90% specificity. Once again, this is an improvement with respect to CA19-9 (p = 0.0082, one-sided bootstrap test) taken as a univariate model developed in the discovery set; this CA19-9 model predicted PDAC status with an AUC of 0.80 (95% CI 0.66–0.93), sensitivity 0.65 (95% CI 0.48–0.56), PPV 0.49 (95% CI 0.41–0.56) and NPV of 0.95 (95% CI 0.92–0.98) at 90% specificity in the validation set. If we further validate only on the benign disease controls and PDACs collected from ADEPTS, the diagnostic-specific ensemble signature achieved an AUC of 0.96 (95% CI 0.92–0.99), sensitivity 0.82 (95% CI 0.64–0.95) at 90% specificity. This performance is also significantly higher than the performance of CA19-9 in a univariate model: AUC of 0.79 (95% CI 0.64–0.93) (p = 0.013 when compared with the full signature, one-sided test) and sensitivity of 0.18 (95% CI 0.03–0.69).

A closer examination of the individual performances of each base-learner classifier (Fig 2A and 2B) reveals that the logistic regression stacked ensemble approach has superior performance in both discovery and validation sets. Despite the best base-learner being trained on samples diagnosed as ’Gastritis/Reflux Disease’ (Fig 2A and 2B), its performance was also superseded by the AUC computed with the stack model, the logistic regression coefficients of which are delineated in Table E in S1 Appendix. The stack model significantly relies on the “Healthy”, “Chronic Pancreatitis”, “IgG4 Disease”, “Irritable Bowel Syndrome”, ‘Other Biliary Duct Disease”, “Sphincter of Oddi Dysfunction”, “No Relevant Diagnosis”, “Other Cancer” and “Pancreatic Cyst” base-learners. Even though the remaining diagnostic class base-learners, including "Gastritis/Reflux Disease", did not reach statistical significance (p<0.05), employing a stack that solely resorts to significant base-learners led to a reduction in generalization capacity: AUC 0.98 (95% CI 0.97–0.99), sensitivity 0.98 (95% CI 0.95–1), PPV 0.92 (95% CI 0.91–0.92), NPV 0.97 (95% CI 0.94–0.99) in the discovery set; AUC 0.93 (95% CI 0.87–0.99), sensitivity 0.82 (95% CI 0.64–0.95), PPV 0.53 (95% CI 0.47–0.57), NPV 0.97 (95% CI 0.95–0.99) in the validation set. Although the differences are not substantial, we retain the full set of base-learners to enhance the generalization capacity for predicting PDAC in unseen data sets and new samples. In fact, upon following recursive base-learner elimination the best ensemble was always proven to be the full set of 16 base classifiers.

The employment of stacked diagnosis-specialized classifiers surpassed the AUC performance of state-of-the-art algorithms such as random forests (RRF) and extreme gradient boosting methods (xgbTree), in terms of AUC, sensitivity, positive predictive value, and negative predictive value at 90% specificity (Fig 2C and 2D); although the performance AUC of the stacked classifier was only marginally significantly higher than that obtained with RRF (p = 0.040, one-sided) and not significant when compared with xgbTree (p = 0.26, one-sided), the sensitivity values at 90% specificity obtained with the alternative methods were, in fact, significantly lower, p = 0.028 and p = 0.045, respectively. The ensemble also outperformed a logistic regression model with recursive feature elimination (Fig 2C and 2D, and Materials and methods section) that did not rely on ensemble modelling (p = 0.0066, one-sided), further substantiating our choice of machine learning paradigm for facilitating the identification of PDAC cases in a clinical setting where confounding diagnoses may be present, and the prevalence is low.

The results with additional subsampling algorithms, i.e., under-sampling of the majority class and SMOTE (see Materials and methods), further reinforce our choice (Table F in S1 Appendix). Only xgbTree benefits from SMOTE but the result is not consistent between the discovery and the held-out validation sets when we evaluate the positive predictive value. Moreover, synthetic sample generation has been proven to be less efficient in high-dimensional datasets [41]. Further alternatives with adaptive synthetic data generation are necessary to evaluate the advantages of SMOTE in the current problem [42]. For the sake of simplicity, we generated all the subsequent results with the ensemble of base-learners derived with over-sampling of the minority class, which generalises better in the held-out validation set (Fig 2).

**Fig 3:**
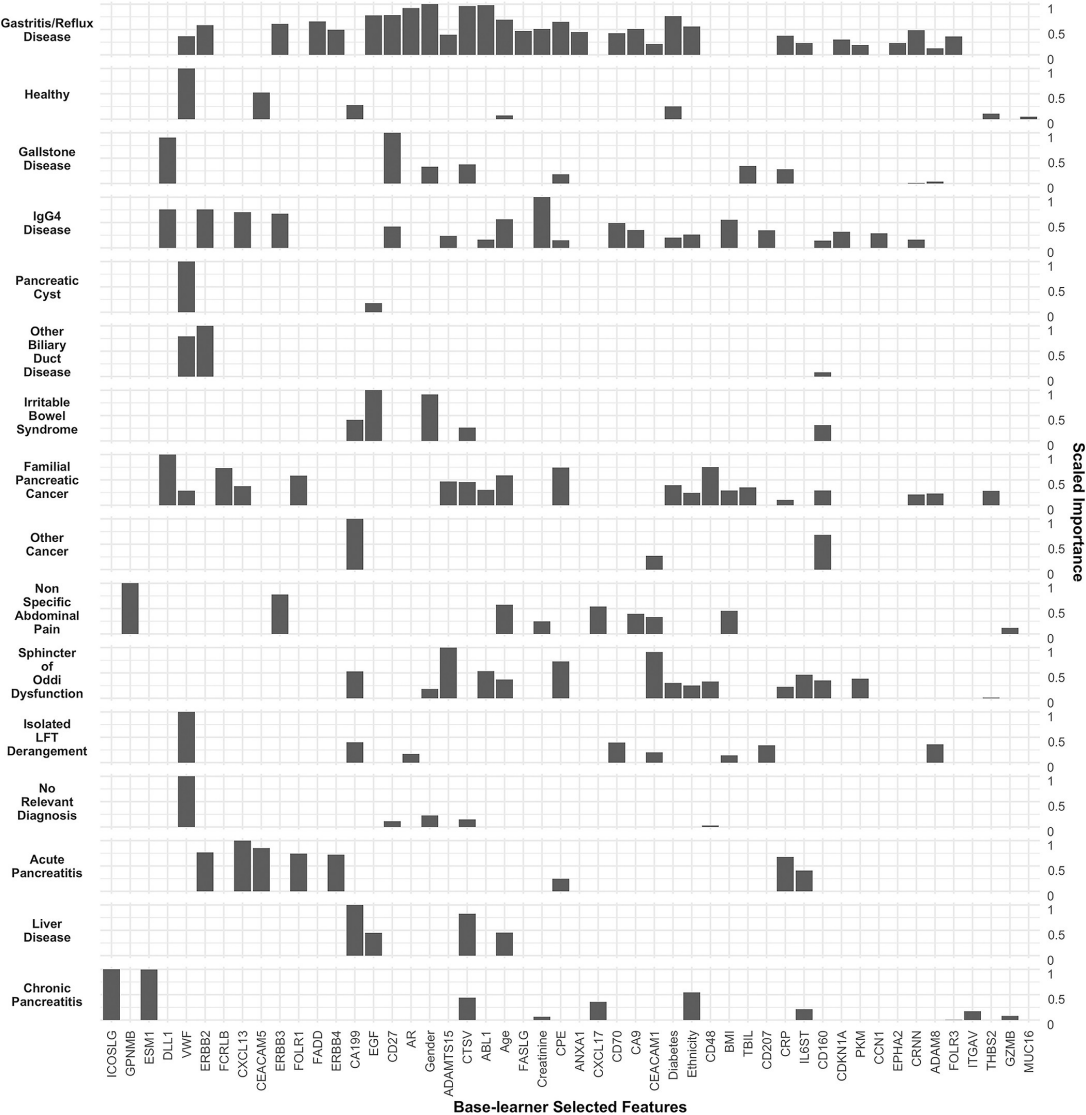
Features selected per diagnosis class (base-learner classifiers). The scaled importance is calculated within each base-learner (Fig 2A). Selected features are ranked from left to right according to the average scaled importance across base learners. See Fig 1 and Tables B, C and D in S1 Appendix for the univariate predictive performances of each of the markers in the discovery and validation sets. See Materials and methods section for details on model-agnostic algorithm for feature importance calculation. See S1 Data file for the underlying data for the figure.

The comprehensive index signature, incorporating all diagnostic categories, was constituted by 49 features, of which 44 were proteins (see Fig 3 for the importance associated with each). Among these proteins, 21 demonstrated a significant association with PDAC in the discovery set; ICOSLG, GPNMB, ESM-1, DLL1, VWF, ERBB2, FCRLB, CEACAM5, EGF, CTSV, FASLG, Creatinine, CPE, CA9/CAIX, TBIL, CD207, CRP, CDKN1A, EPHA2, ITGAV, and MUC-16 (see Fig B and Table C in S1 Appendix). The remaining 23 proteins, namely CXCL13, ERBB3, FOLR1/FR-alpha, FADD, ERBB4, CD27, AREG/AR, ADAM-TS-15, ABL1, ANXA1, CXCL17, CD70, CEACAM1, CD48, IL6ST, CD160, PKM/PKM2, CYR61/CCN1, CRNN, ADAM-8, FOLR3/FRgamma, THBS2, GZMB, did not demonstrate a significant association with PDAC in univariate models (see Fig B and Tables C and D in S1 Appendix). Additionally, five clinical covariates—Gender, Age, Ethnicity, Diabetes, and Body Mass Index (BMI)—were identified as important predictors following comprehensive recursive feature elimination during cross-validation (Fig 3).

Gene Ontology (GO) and biological pathway enrichment (Kyoto Encyclopaedia of Genes and Genomes; KEGG, Reactome Pathway Database; REAC and WikiPathways; WP) analysis was performed for the selected set of features using the *gprofiler2 R package* (Fig C in S1 Appendix). Top significant terms for biological processes (BP) included ‘circulatory system development’, ‘blood vessel morphogenesis’, ‘cell adhesion’, ‘angiogenesis’, ‘blood vessel development’, ‘regulation of cell adhesion’, ‘positive regulation of cell population proliferation’, ‘cell-cell adhesion’, and ‘regulation of developmental process’. Top relevant biological pathways included: ‘PI3K-KAT signalling pathway’, ‘ERBB signalling pathway’, ‘pathways in cancer’, ‘proteoglycans in cancer’, ‘platinum drug resistance’, ‘prostate cancer’, ‘type I diabetes mellitus’, ‘MAPK signalling pathway’ and ‘focal adhesion’.

The scaled importance of each feature and diagnostic class/classifier is depicted in Fig 3. It is of significance to note that not every biomarker was selected by each individualized classifier, highlighting the requirement for an array of diverse predictors, each tailored to specific underlying conditions, to effectively identify PDAC. This is consistent with the idea that heterogeneous ensembles are fundamental for predictive capacity in blind datasets [37,43].

Of the five selected clinical covariates, only Age, Ethnicity, and Gender manifested as significant predictors of PDAC in the validation set, as illustrated in Fig 1 and explained in the data set characteristics subsection (see also Tables A and B in S1 Appendix). It is worth emphasizing that the lack of significant association between certain markers and PDAC in the discovery set does not preclude their inclusion in the signature. These variables were selected due to their contribution to the enhanced robustness and generalization capacity in predicting PDAC during cross-validation with a recursive feature elimination routine (see Materials and methods). A similar trend was verified in prior work focussed on ensemble models for PDAC early detection against healthy controls and further substantiates the need for extensive discovery analysis in the data sets collected for the present project [37].

In our comprehensive analysis, we identified eight features that exhibited relatively elevated scaled importance in distinguishing controls from patients diagnosed with PDAC. These features, as detailed in Fig 3, include the biomarkers CA19-9, VWF, CPE, CTSV, CEACAM1, and CD160. To rigorously assess the diagnostic utility of these selected features, we devised a reduced index employing the diagnosis-specific ensemble strategy previously delineated. Diabetes and Age were incorporated as clinicodemographic variables. This strategy facilitates a targeted evaluation of the features’ collective performance in a clinical context. Detailed performance metrics and analytical outcomes of this reduced index are presented in S1 Appendix and Table 2.

**Table 2:** Performance summary for selected models in symptomatic patients. The probability values used to calculate the performance metrics were generated with each model developed in the training set and reported in the main text. Probability values for symptomatic patients belonging to the training set and validation set were concatenated to generate the ROC curves. Only ADEPTS samples had symptoms information. A. L. Derang.: Asymptomatic LFT Derangement. B. Pain: Back Pain. C. B. Habit: Change in Bowel Habit. W. Loss: Weight Loss. 95% confidence intervals are provided in parentheses. See also Table H in S1 Appendix for the explicit performance ranks according to model, symptom and metric and Fig 5. *Receiver Operating Characteristic (ROC) Area Under the Curve (AUC), Sensitivity (Sens), Positive Predictive Value (PPV) and Negative Predictive Value (NPV) at 90% Specificity (Spec).*

Models	Metric	Symptom (Yes)				
		A.L.Derang.	A. Pain	A. B. Habit	W.Loss	Jaundice
CA19-9	ROC	0.85 (0.62–1)	0.81 (0.65–0.96)	0.82 (0.57–1)	0.74 (0.58–0.90)	0.70 (0.53–0.86)
	Sens90	0.75 (0.38–1)	0.69 (0.44–0.94)	0.71 (0.42–1)	0.53 (0.24–0.76)	0.41 (0.14–0.73)
	PPV90	0.54 (0.37–0.61)	0.36 (0.26–0.43)	0.46 (0.34–0.55)	0.70 (0.50–0.77)	0.83 (0.64–0.90)
	NPV90	0.96 (0.90–1)	0.97 (0.95–0.99)	0.96 (0.93–1)	0.81 (0.73–0.90)	0.55 (0.46–0.73)
Index signature	ROC	0.98 (0.95–1)	0.98 (0.97–1)	0.97 (0.92–1)	0.95 (0.90–1)	0.89 (0.79–0.99)
	Sens90	1 (0.62–1)	0.94 (0.81–1)	0.86 (0.56–1)	0.94 (0.29–1)	0.73 (0.36–0.91)
	PPV90	0.61 (0.49–0.61)	0.43 (0.40–0.45)	0.51 (0.41–0.55)	0.8 (0.56–0.81)	0.90 (0.82–0.92)
	NPV90	1 (0.94–1)	0.99 (0.98–1)	0.98 (0.95–1)	0.97 (0.75–1)	0.73 (0.54–0.89)
Reduced signature	ROC	0.97 (0.93–1)	0.92 (0.88–0.99)	0.91 (0.83–0.98)	0.92 (0.85–0.99)	0.82 (0.67–0.97)
	Sens90	1 (0.63–1)	0.81 (0.38–1)	0.57 (0.14–1)	0.71 (0.35–0.94)	0.77 (0–0.95)
	PPV90	0.61 (0.49–0.94)	0.40 (0.23–0.45)	0.41 (0.15–0.55)	0.75 (0.6–0.8)	0.90 (0–0.92)
	NPV90	1(0.94–1)	0.98 (0.95–1)	0.95 (0.9–1)	0.88 (0.76–0.97)	0.76 (0.42–0.94)

In evaluating the efficacy of our ensemble modelling approach and data analysis protocol, we benchmarked it against biomarker combinations reported in existing literature [40,44–48]. Our comparison pitted our panels and protocol against a recursive feature elimination routine presented above that did not resort to ensemble modelling (RFE glm in Fig 2); the latter selected Diabetes, ABL1, ERBB3, ESM1, EGF, SYND1, PPY, TGFA, VEGFA as the best feature combination. From this set the first five markers were involved in the diagnosis-specific ensemble index (Fig 3), and from the remaining only TGFA and VEGFA are reported in the literature as part of best performing panels [40,44–48]. Despite this overlap, given that the starting point for RFE glm was the same as in our ensemble approach, a greater portion of single markers reported in the literature should have arisen as the most competitive when applied to our data set. This is not verified, performance wise, since the diagnosis-specific ensemble modelling still outperformed any other alternative, thus confirming our protocol’s added value in distinguishing diagnosed PDACs from benign and healthy controls (Fig 2).

Furthermore, it is critical to acknowledge that the best-performing index detailed in this study significantly deviates from our earlier work [37]. In the previous study, the goal was to develop a combined index that could distinguish undiagnosed PDAC cases from healthy controls years before a cancer diagnosis was made. In contrast, the current study focuses on identifying an analysis strategy or pipeline leading to effective biomarker panels for use in secondary care among at-risk populations. This shift in focus reflects our ongoing efforts to refine diagnostic tools tailored to the specific needs of different clinical contexts.

### Application of the full PDAC ensemble signature in symptomatic patients

Our subsequent aim was to explore whether specific clinical manifestations were correlated with PDAC status in our ADEPTS patient cohort, for which such information was available (refer to Fig D and Table G in S1 Appendix). As a similar type of data was not available for the UKCTOCS subset (healthy controls) used in this work, we focused this section on the ADEPTS cohort.

**Fig 4:**
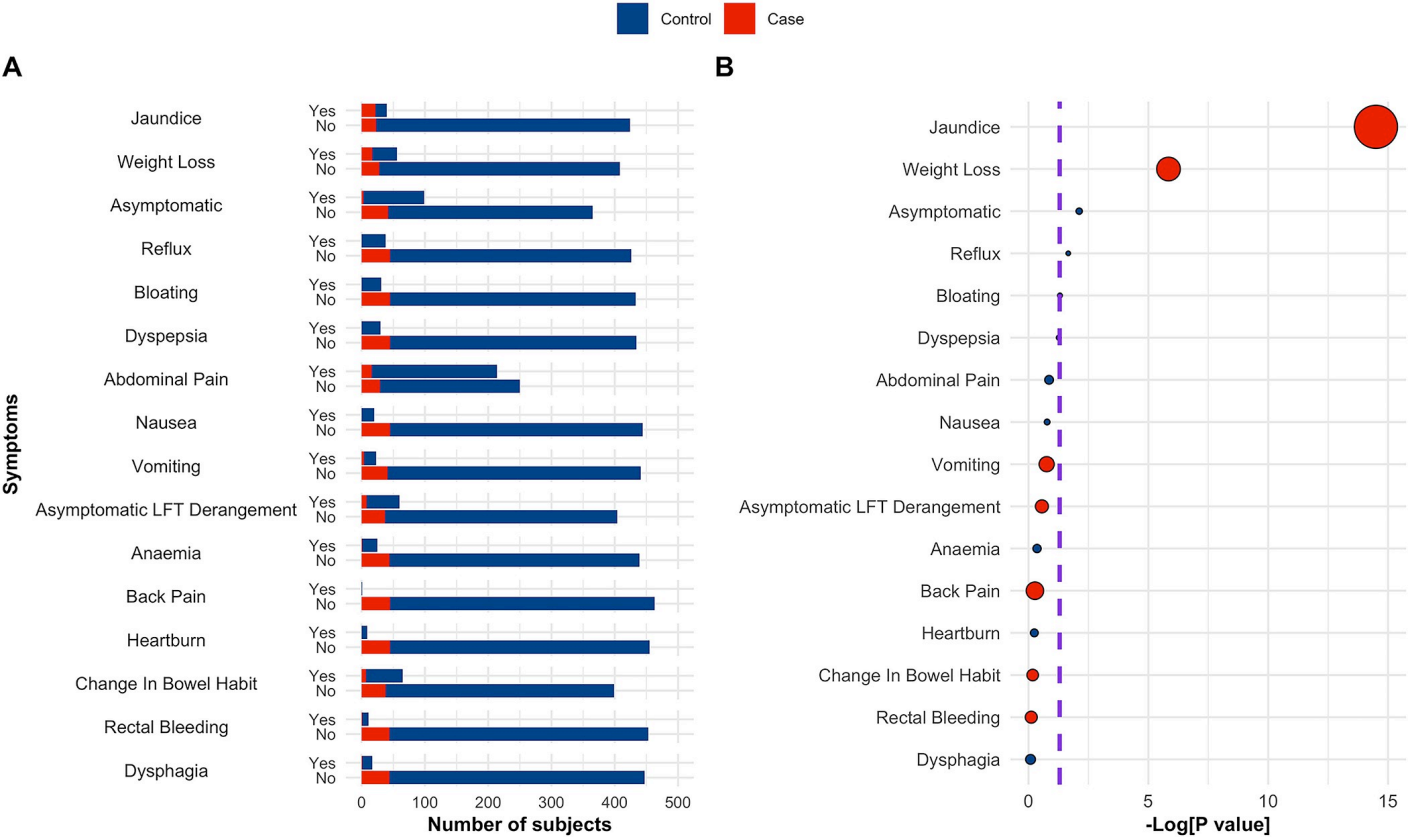
Association between symptoms and PDAC. **A** Number of subjects with each symptom according to PDAC status, case or control**. B** Association of symptoms with PDAC status, p values were calculated according to a logistic regression model with a bias reduction method. Purple dashed lines correspond to -Log [0.05]. In B dot sizes correspond to odds ratios and are colour coded according to their respective values, i.e., blue if OR<1 and red if OR>1. See also Table I in S1 Appendix. Only samples belonging to the ADEPTS cohort were used as no information about symptoms was available for the UKCTOCS set of samples.

In our prior research, we analysed 12 "red-flag" symptoms reported by patients up to 22 months before the diagnosis of pancreatic cancer was established [17]. In this work, ‘Vomiting’ (p = 0.17), ‘Asymptomatic LFT Derangement’ (p = 0.28), ‘Back pain’ (p = 0.54), ‘Change in Bowel Habit’ (p = 0.67) and ‘Rectal Bleeding’ (p = 0.76) were selected for PDAC (versus benign disease controls), yet only ‘Jaundice’ (p = 3.22×10^−15^), and ‘Weight Loss’ (p = 1.44×10^−6^) were significantly associated with PC cancer cases in the set of samples randomly selected from the ADEPTS cohort, in which the biomarker panel was tested (Fig 4B and Table G in S1 Appendix). Unsurprisingly, ‘Reflux’ (p = 0.022) and ‘Bloating’ (p = 0.048) were significantly associated with benign controls. Interestingly, ’Abdominal Pain’, ’Heartburn’, ’Anaemia’, and ’Dysphagia’ upon presentation were aligned more with the benign control cohort, albeit not significantly (refer to Fig 4 and Table G in S1 Appendix).

**Fig 5:**
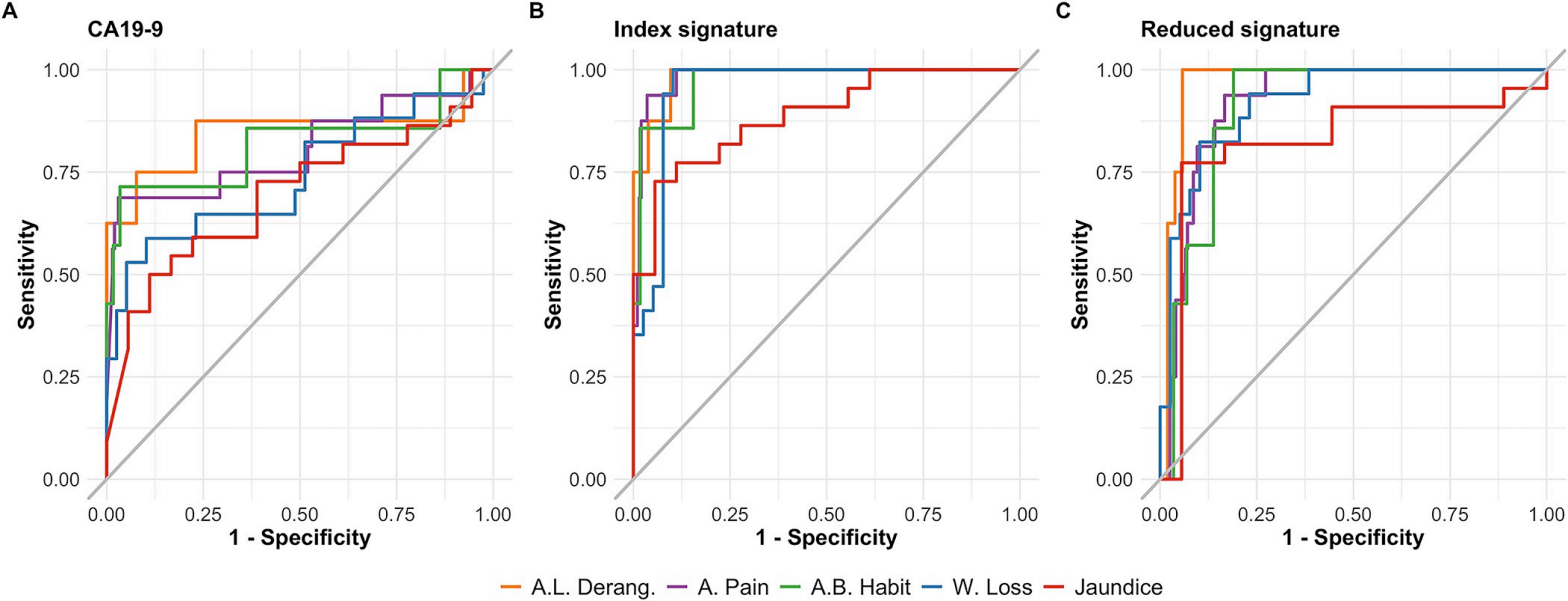
Receiver operating characteristic curves for selected models in symptomatic patients. **A** Only CA19-9. **B** Full index signature. **C** Reduced index signature. The probability values used to calculate the performance metrics were generated with each model developed in the discovery set and reported in the main text. Probability values for symptomatic patients belonging to the discovery set and validation set were concatenated to generate the ROC curves. Only ADEPTS samples had symptoms information. A. L. Derang.: Asymptomatic LFT Derangement. B. Pain: Back Pain. C. B. Habit: Change in Bowel Habit. W. Loss: Weight Loss. See also Table 2 for numerical values for area under the curve and other metrics.

Within the framework presented in preceding sections, our ensemble of classifiers was developed independently of symptomatic data. To assess the overall efficacy of our signature and its predictive capacity for PDAC, we scrutinized its performance on a subset of ADEPTS patients, belonging to both discovery and validation cohorts, manifesting with ’Weight Loss’ (n = 56) and ’Jaundice’ (n = 40) (refer to Fig 4 and Table G in S1 Appendix). For each sample within these cohorts, where symptom data was accessible, probability scores were derived based on the ensemble model formulated above. We should emphasize that no additional model refinement was pursued and a simple concatenations of probability outputs was done. The decision to aggregate these probability scores is further rationalized by the relatively limited patient count exhibiting ’Weight Loss’ and ’Jaundice’ within the individual discovery and validation datasets (Table G in S1 Appendix). In the ADEPTS subset of samples presenting with ’Weight Loss’, an AUC of 0.95 (95% CI 0.90–0.1), a sensitivity of 0.94 (95% CI 0.29–1), a PPV of 0.80 (95% CI 0.56–0.81), and a NPV of 0.97 (95% CI 0.74–1) at 90% specificity were achieved (Fig 5 and Table 2). In patients presenting with ’Jaundice’, an AUC of 0.89 (95% CI 0.79–0.99), a sensitivity of 0.73 (95% CI 0.36–0.91), a PPV of 0.90 (95% CI 0.82–0.92), and a NPV of 0.73 (95% CI 0.54–0.89), at 90% specificity, were observed (Fig 5 and Table 2). Compared with the AUC obtained with a simple CA19-9 logistic regression model developed in the discovery set and by concatenating the probability scores in the discovery and validation as done above, a significantly lower AUC of 0.74 (95% CI 0.58–0.90) is achieved (p = 1.29×10^−11^, one-sided bootstrap test), with a sensitivity of 0.53 (95% CI 0.24–0.76), a PPV of 0.70 (95% CI 0.50–0.77), and a NPV of 0.81 (95% CI 0.73–0.90), at 90% specificity, for patients presenting with ’Weight Loss’. For patients presenting with ‘Jaundice’ an AUC of 0.70 (95% CI 0.53–0.86) is reached, also significantly inferior (p = 1.94×10^−7^), with a sensitivity of 0.41 (95% CI 0.14–0.73), a PPV of 0.83 (95% CI 0.64–0.90), and a NPV of 0.55 (95% CI 0.46–0.73), at 90% specificity, for CA19-9 as the single predictor.

With respect to other non-localising symptoms of note (Fig 5 and Tables 2 and H in S1 Appendix), the best predictive performance was noted for the full index signature where it was able to differentiate patients presenting with ‘abdominal pain’ due to benign conditions vs. PDAC with an AUC of 0.98 (95% CI 0.97–1), sensitivity of 0.94 (95% CI 0.81–1), PPV 0.43 (95% CI 0.40–0.45) and a NPV of 0.99 (95% CI 0.98–1), at 90% specificity. In those presenting with ‘change in bowel habit’, an AUC of 0.97 (95% CI 0.92–1), sensitivity 0.86 (95% CI 0.81–1), PPV of 0.51 (95% CI 0.41–0.55) and NPV of 0.98 (95% CI 0.95–1) was obtained. Both the full ensemble index and the 8-marker signature showed superior predictive performance to CA19-9 as a single marker (see Table I for the respective p-values, in S1 Appendix).

### Correlation of the full PDAC ensemble signature with QCancer pancreatic score

In our final analysis, we juxtaposed the performance of our full ensemble classifier PDAC index against the QCancer risk prediction index, a clinical decision support tool available for primary care physicians, that integrates a myriad of individual-specific risk factors including age, sex, ethnicity, clinical measurements, diagnoses, and patient-reported symptoms into a risk stratifying point of care questionnaire [21]. The ‘Today’s QCancer’ index evaluates an individual’s current risk of having an undiagnosed cancer as well as the specific risk for 9 distinct underlying cancer types, including pancreatic (‘pancreatic’ score) [49,50]. The aim was to determine whether in combination, the QCancer eCDST and our biomarker index signature would be able to better discriminate PDAC patients in a symptomatic (ADEPTS) cohort or whether it would be redundant. As the current risk threshold set by the NICE is at 3% for triggering specialist referrals [51], we opted to assess the combined performance of our index signature and the eCDST at a same or lower cut-off values.

**Fig 6:**
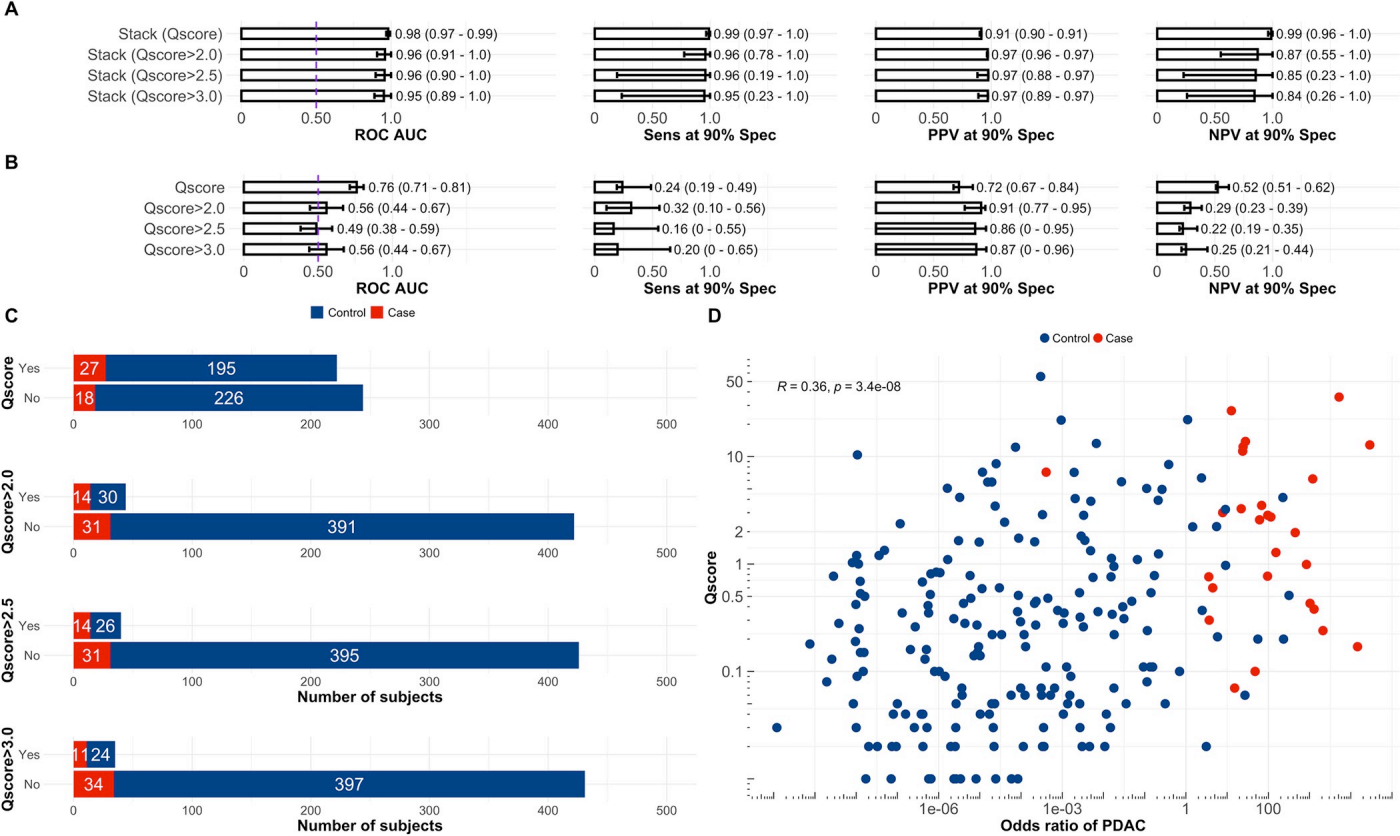
Prediction of PDAC in patients with specific symptoms and according to QCancer score values. The ensemble stack was selected as the best model according to Fig 2. **A** Performance of the stack in participants for which a Qscore had been calculated or above a specific threshold, bigger than 2, 2.5 or 3.0. **B** Performance of the Qscore taken as the predictor of PDAC risk in participants for which a Qscore had been calculated or above a specific threshold, bigger than 2, 2.5 or 3.0. **C** Number of subjects that had a calculated Qscore or are above a specific threshold, bigger than 2, 2.5 or 3.0**. D** Correlation between QCancer score and odds ratio of PDAC according to the stacked ensemble. D is in log scale and R stands for the Person correlation coefficient and p for the p-value calculated with a t-test. The QCancer score is identified as Qscore in the figure panels. *Receiver Operating Characteristic (ROC) Area Under the Curve (AUC), Sensitivity (Sens), Positive Predictive Value (PPV) and Negative Predictive Value (NPV) at 90% Specificity (Spec).*

The number of samples for which a QCancer score was computed is illustrated in Fig 6C. Using the diagnostic-specific ensemble model delineated previously, probability scores for samples in both discovery and validation cohorts were used to ascertain the combined ROC AUC for those samples possessing a QCancer score. This amalgamation was imperative, considering the reduced number of samples with an associated QCancer score (Fig 6C). It should be emphasized that no subsequent refinements or training of the algorithm were conducted. The ensemble stack index demonstrated a remarkable performance, achieving an AUC of 0.98 (95% CI 0.97–0.99), a sensitivity of 0.99 (95% CI 0.97–1), a PPV of 0.91 (95% CI 0.90–0.91), and a NPV of 0.99 (95% CI 0.96–1), at 90% specificity. Interestingly, when considering only samples with a QCancer risk above 2 or 2.5, the biomarker and clinical covariate ensemble index exhibited comparatively lower performance (Fig 6A). For a QCancer risk above 3.0, the performance of the index decreases minimally once again, which is expected as the difficulty of correctly singling out cases from confounding controls is increased (Fig 6A). However, the QCancer pancreatic score did exhibit a correlation with the odds of PDAC as determined by the ensemble classifier (R = 0.36, p = 3.4×10^−8^, Fig 6D) which highlights an important link between the purely clinical variables recorded for this cohort and the PDAC signature. Most importantly, the stacked index succeeded in attributing higher odds ratios above 1 to several PDAC cases that would have otherwise escaped detection had a QCancer score above 3 been taken as the risk predictor (Fig 6D). Contrarily, when depending exclusively on the QCancer score, and using it to calculate the ROC AUC, the predictive capacity for PDAC in the ADEPTS samples is noticeably diminished in comparison to the performance of the ensemble index (Fig 6B); this was verified in all samples with a Qcancer score (p = 1.56×10^−18^, one-sided bootstrap test comparing AUCs), with a score above 2 (p = 1.24×10^−10^), above 2.5 (p = 3.68×10^−13^) and above 3 (p = 2.33×10^−8^). This justifies the PDAC signature as a useful complementary resource for enhanced and accelerated diagnosis in the clinic.

## Discussion

Our objective was to derive a data analysis strategy and construct a multi-biomarker signature that could effectively differentiate individuals with non-specific yet concerning symptoms attributable to both benign abdominal pathologies and PDAC. CA19-9 tumour marker blood levels are currently used clinically to help confirm PDAC diagnosis in a clinical context (positive findings on imaging, histopathology), prognosticate and monitor recurrence following tumour resections [31]. Its absent expression in Lewis body negative blood group individuals, an overall limited predictive capacity (79–81% test sensitivity and 82–90% specificity at best), especially in the presence of certain inflammatory pancreatico-biliary conditions, have driven researchers to rather combine it in multi-marker panels to enhance its predictive performance [26,27,31]. In an evolving multi-omics area, reported panels have included proteins, circulating nucleic acids (micro-RNA, cfDNA) or tumours cells, metabolites, and products of alternative DNA splicing and methylations [31,52], developed to differentiate PDAC from healthy controls and those with benign pathologies. Yet, the role of such diagnostic and screening panels in symptomatic cohorts remains unestablished.

The majority of the sampled population in our study is an enriched, symptomatic, secondary care cohort where the prevalence of PDAC was close to 8%, representing figures observed in our hepatobiliary specialised referral centres. By using this target population and their unique set of serum samples provided by the ADEPTS study [44], we were able to develop a biomarker signature in a cohort of patients who were referred to our participating centres (University College London Hospitals, London UK and the Royal Free Hospital, London UK) with various abdominal and hepatobiliary conditions which in symptomatic presentation might overlap with PDAC [17]. Moreover, we included samples from patients with known risk factors for PC (chronic pancreatitis, those with family history of PDAC and cystic lesions of the pancreas, CLPs) and with biliary conditions that are known confounders of CA19-9 (i.e. biliary tract inflammation/obstruction, pancreatitis, CLPs)—the only tumour marker clinically applied in the workup and management [26,48,53] of PDAC.

We employed ensemble learning methods, which have achieved impressive accuracy in numerous complex classification tasks [37,43,54,55]. Specifically, we utilized stacking—a form of meta-learning [43]—to create a superior-level predictive model based on the predictions of diagnosis-specific base classifiers. These classifiers leveraged a diverse set of features, highlighting the fundamental importance of heterogeneity arising from specific diagnoses when compared against PDAC, an approach previously demonstrated to be effective [37,54]. Moreover, this study enabled us to evaluate the specificity of our general early detection machine learning approach [37] within a relevant symptomatic population, thereby allowing us to address confounding factors that may impact their performance. The use of such diagnostic specialized base-learners was further justified by the data asymmetry between PDAC cases and controls observed in both the discovery and held-out validation datasets.

Across all diagnosis classes (base learners) the ensemble index signature which comprised 44 clinical and serum protein covariates predicted PDAC (all stages) with an AUC of 0.98 (95% CI 0.98–0.99); at 90% specificity, a sensitivity of 0.99 (95% CI 0.98–1), PPV 0.92 (95% CI 0.91–0.92) and NPV 0.99 (95% CI 0.97–1) was reached, in contrast to CA19-9 as a single predictor under a logistic regression model—AUC 0.79 (95% CI 0.66–0.91), sensitivity 0.67 (95% CI 0.50–0.83), PPV 0.32 (95% CI 0.26–0.38) and NPV of 0.97 (95% CI 0.96–0.99). On validation, an AUC of 0.95 (95% CI 0.91–0.99), sensitivity 0.86 (95% CI 0.68–1), PPV 0.54 (95% CI 0.48–0.58) and NPV of 0.98 (95% CI 0.95–1) was achieved by the signature, compared to an AUC of 0.80 (95% CI 0.66–0.93), sensitivity 0.65 (95% CI 0.48–0.56), PPV 0.49 (95% CI 0.41–0.56) and NPV of 0.95 (95% CI 0.92–0.98), for CA19-9. The ensemble panel also outperformed other state-of-the-art methods. Since these alternative methods also rely on the same starting set of features and follow standard approaches that led to previously reported panels by other researchers, we must conclude that our proposed index also showed better performances in our data than other published combinations of biomarkers.

The performance of this index panel and model development methodology must be appreciated within the context of the complex biology associated with each of the ensembled diagnostic classes, i.e., the challenges associated with biomarker alterations on the background of pancreatico-biliary inflammatory and obstructive pathologies. When applying a redacted, 8-marker signature (CA19-9, VWF, CPE, CTSV, CEACAM1,CD160, Diabetes and Age)—features that were selected with relatively high importance across most base learners, the performance was naturally reduced, yet still performed significantly better against CA19-9 as a single marker during discovery. Using the general linear model stack as was done in the case of the full index, the reduced signature predicted PDAC with AUC of 0.97 (95% CI 0.95–0.98), sensitivity 0.98 (95% CI 0.95–1), PPV 0.92 (95% CI 0.91–0.92) and NPV of 0.98 (95% CI 0.94–1), at 90% specificity (Fig EC in S1 Appendix), values comparable to the full index. During validation, however, the predictive capacity of the reduced signature was significantly reduced compared to the full stacked model (Fig ED in S1 Appendix) and only marginally superior to CA19-9 alone across the cohort. In contrast, it still outperformed CA19-9 by a significant margin when predicting PDAC against healthy controls (supplementary text in S1 Appendix).

As validation of its performance, we also applied the full ensemble index signature to the cohort when re-stratified based on presenting symptoms, with no further model refinement. Since the ensemble of classifiers were developed independently of symptomatic data, the aim was to test the signature performance in differentiating PDAC cases from controls by accounting for presenting symptoms which have been linked with repeated primary care consultations up to two-years prior to PDAC diagnosis [17] (Fig 4). Enriched by fulfilling certain sociodemographic, clinical and attributable suspicious symptoms (identified using eCDSTs such as QCancer tool), symptomatic patients would form an ideal cohort for further risk stratification by minimally invasive blood biomarker testing for prioritisation of more invasive (and costly) investigations. Yet, contrary to the full ensemble index, in the cohort used in the current work the QCancer score used as the sole predictor of PDAC did not achieve significant performances in samples above the threshold of 3%. This further motivates the recourse to combined strategies where complementary biomarker panels such as those identified by ensemble modelling approaches could improve early detection when used in conjunction with eCDSTs.

In our test subjects, however, only ‘Jaundice’ (p = 3.22×10^−15^), and ‘Weight Loss’ (p = 1.44×10^−6^) were significantly associated with PDAC. When testing the diagnostic performance of the full index signature in all symptomatic patients presenting with ‘Weight Loss’, the signature significantly outperformed CA19-9: AUC_signature_ of 0.95 (95% CI 0.90–0.1) vs. AUC_CA19-9_ of 0.74 (95% CI 0.58–0.90) (Fig 5A and Tables 2 and I in S1 Appendix). ‘Weight loss’ has previously been reported to have the longest diagnostic interval in a prospective primary cohort study (SYMPTOM pancreatic study), assessing symptom trends and associated diagnostic intervals in PC [11]. Attesting to the full index signature’s capacity as a rule out test in such patients, is its outstanding negative predictive value compared to that of CA19-9 (0.97 95% CI 0.75–1 vs. 0.81 95% CI 0.73–0.9, respectively) (Table 2). Similarly, the index signature performed superiorly to CA19-9 in jaundiced patients (AUC of 0.89 (95% CI 0.79–0.99) vs. CA19-9 AUC of 0.70 (95% CI 0.53–0.86), see also Table I in S1 Appendix), which underscores once again the increased capacity of the ensemble index to better identify PDAC in the presence of a known confounder of CA19-9 [26,48].

While our study provides valuable insights, it is not without limitations. While the observed prevalence of PDAC in this study aligns with secondary care population trends, enhanced specificity and positive predictive value would necessitate larger cohorts with an increased number of cases. Moreover, the sample set representing the ’Healthy’ control class warrants expansion to incorporate a more diverse population of both men and women. This control class, derived from the UKCTOCS samples used in a previous study [37], was exclusively comprised of women. Given its superior performance in predicting PDAC, as depicted in Fig 2, the inclusion of male samples within this class could further enhance the breadth of the panel of markers identified in this study. Lastly, although diabetes emerged both as a risk factor and a central clinical covariate in our signature (including in the reduced panel), we must emphasize and recognise the lack of complete (type, duration) data in the UKCTOCS cohort [37]. Nevertheless, diabetes mellitus (and in particularly of new onset) is an established risk factor and therefore its inclusion as a relevant feature in the signature is of no surprise [6].

While our index was superior in its predictive performance to CA19-9 alone and other biomarker combinations reported in the literature, in addition to compensating for asymmetric binary classes by creating a diagnostic-specific ensemble, its complexity challenges its utilisation in clinic. Yet, in the current era of rapidly evolving assay technologies, the utilization of a complex biomarker signature comprising numerous variables has gained significant relevance. While the complexity of these biomarker signatures may pose analytical challenges, the evolving assay technologies offer the means to effectively harness their potential.

Future enhancements however, will naturally necessitate the study of larger cohorts and multi-modal data, potentially incorporating a biomarker-contextualized machine learning perspective that accounts for sample-specific aspects related to diagnosis, a strategy employed in other cancer research domains [56]. The utilization of disease trajectory tracking and clinical history analysis [57] may also facilitate the application of advanced deep learning techniques and electronic health data. When combined with ensemble biomarker signatures taken for example in a longitudinal context [37,58], these approaches could enhance the estimation of PDAC risk within an enriched symptomatic population.

## Materials and methods

### Ethics statement

For the Accelerated Diagnosis of neuro Endocrine and Pancreatic TumourS (ADEPTS) study, University College London (UCL)/ University College London Hospital (UCLH) Research Ethics Committee reference 06/Q0512/106, IRAS Number 234637, NIHR portfolio no. 7343, patients were recruited at gastroenterology/hepatobiliary and surgical clinics at UCLH and the Royal Free Hospitals (RFH), London, UK. All patients recruited to the ADEPTS study provided written informed consent and no data allowing identification of patients was used.

For the UK Collaborative Trial of Ovarian Cancer Screening (UKCTOCS), Joint UCL/UCLH Research Ethics Committee A (Ref. 05/Q0505/57), written informed consent for the use of samples in the trial and secondary ethically approved studies was obtained from donors and no data allowing identification of patients was used.

### Study design

As our cohort, we used serum samples from the ADEPTS study [44] aimed at detecting pancreatic cancer in patients at an earlier stage. As part of the Early Diagnosis Research Alliance, the ADEPTS study (previously referred to as TRANSlational research in BILiary tract and pancreatic diseases study), commenced in 2018 and included a multicentre prospective blood sample collection from patients with non-specific but concerning symptoms associated with PDAC. Patients were recruited at gastroenterology/hepatobiliary and surgical clinics at UCLH and the RFH, London, UK. Blood samples were collected from subjects with benign hepatobiliary conditions as well as those with PDAC (stages I-IV).

For PDAC patients, tumour staging was performed according to the AJCC 8^th^ edition (TNM) based on cross-sectional imaging and for those undergoing surgery, based on multi-disciplinary team recordings. All included PDAC cases were histologically confirmed by UCLH and RFH local pathologists based on tissue analysis obtained by endoscopic ultrasound guided fine needle biopsies or specimens obtained during surgical resection.

For benign disease controls, patients were selected to include the following diagnoses: chronic pancreatitis, intraductal papillary mucinous neoplasms (IPMN), or benign pancreatic diseases (e.g., serous cystadenomas and pancreatic heterotopia). Patients with acute and chronic pancreatitis, pancreatic cysts, benign biliary duct diseases (e.g., IgG4 disease), liver disease, gastritis/reflux disease, gallstones as well as those with familial history of pancreatic cancer, were also used. Samples also included those collected from patients presenting with non-specific symptoms which were not otherwise explained by an underlying gastrointestinal pathology (such as non-specific abdominal pain and irritable bowel syndrome) as well as other malignancies. Medical history and confirmation of diagnosis was obtained from hospital medical records and included GP and secondary clinic referral letters. For 45 patients, a QCancer score was available at time of specialist centre consultations. QCancer calculates the probability of an individual as harbouring an existing, yet undiagnosed cancer, by considering their specific risk factors and presenting symptoms. These are digitally available for primary care physicians through patient record and data management portals such as EMIS Web and INPS and designed as clinical decision support tools to aid in assessment of need for specialist referrals [21–24].

To further represent the healthy population we also used samples from 72 healthy control UKCTOCS [36] samples that were collected from a nested case control discovery study part of UKCTOCS reported before [37]. The original UKCTOCS dataset from which data was used was derived from serum samples collected from post-menopausal women, aged between 50 and 74 years, who were recruited between the years 2001 and 2005 [36]. The collection of these samples was conducted in accordance with a specific Standard Operating Procedure [59,60]. For the current work our interest lies only with the UKCTOCS matched non-cancer controls, i.e., with no cancer registry code, from individual women selected based on collection date, age, and centre to minimize variation due to handling and storage. Comprehensive information regarding diabetes status for the selected UKCTOCS participants was either unavailable or incomplete. In addition, data on disease duration was not accessible. Consequently, it was not feasible to stratify samples to discovery and validation sets based on the type of diabetes they may have had. For the purposes of this study, only healthy controls that were matched to PDAC cases, with less than one year to diagnosis, were utilized.

A total of 539 serum samples (493 controls and 46 PDAC cases, see Table 1) were analysed using the Olink multiplex immunoassay Oncology II panel in addition to five in-house markers: Carbohydrate antigen 19–9 (CA19-9), Interleukin 6 Cytokine Family Signal Transducer (IL6ST/IL6RB), von Willebrand factor (VWF), Pyruvate kinase isozymes M1/M2 (PKM/PKM2) and Thrombospondin 2 (THBS2/TSP2). The selection of additional markers, beyond CA19-9, was informed by our preceding research in early detection of PDAC [37,48]. In those studies, a panel of markers was identified due to its demonstrated ability to facilitate the early detection of pancreatic cancer, with a lead time of up to two years prior to diagnosis.

### Serum analyte measurements

All ADEPTS [44] samples were randomized for testing. Table J in S1 Appendix summarizes dilution factors and coefficients of variation. CA19-9 was measured using the Mucin PC/CA19-9 ELISA Kit (Alpha Diagnostic International) according to the manufacturer, using a 1:4 serum dilution. For VWF, we resorted to the Von Willebrand Factor Human ELISA Kit (abcam) at a 1:100 serum dilution. IL6ST/IL6RB by Quantikine human soluble gp130 (R&D Systems), according to manufacturer recommendations, at a 1:100 serum dilution. THBS2/TSP2 was measured using the Quantikine Human Thrombospondin-2 Immunoassay (R&D Systems) at a 1:10 serum dilution. Pyruvate kinase M2 (PKM2) was measured with an ELISA (Cloud-Clone Corp) at a 1:10 dilution.

We outsourced tests using the multiplex immunoassay Oncology II panel from Olink on all samples. This Olink panel measured known cancer antigens, growth factors, receptors, angiogenic factors, and adhesion regulators (as detailed in Table K in S1 Appendix). Identical assays were performed on a subset of samples derived from the UKCTOCS study [59,60].

To bridge the normalized protein expression values from Olink between the UKCTOCS and ADEPTS datasets, we selected a representative sample set of 16 from each cohort and plated them together. Subsequently, a correction was applied to the datasets using the statistical algorithms recommended in the Olink data normalization white paper [61]. This method ensured that the data from different batches and studies were comparable, thereby enhancing the robustness and validity of the findings.

### Statistical analysis

The selected set of ADEPTS samples used in this work was partitioned into two distinct sets: a discovery subset, comprising two-thirds of the total sample size, and a held-out validation subset, encompassing the remaining one-third. All algorithms were trained in the discovery subset. Allocation into each subset was performed by stratifying for specific age ranges, diabetes status, PDAC status and control diagnosis class. For the PDAC cases, tumour stage was also used. The age stratification ranges were the following: 18<Age≤28; 29<Age≤38; 39<Age≤48; 49<Age≤58; 59<Age≤68; 69<Age≤78; Age≥79. The samples assigned to the control class were made of benign conditions such as: Sphincter of Oddi dysfunction, Pancreatic Cyst, Other Cancer, Other Biliary Duct Disease, No Relevant Diagnosis, Liver Disease, Irritable Bowel Syndrome, IgG4 Disease, Gastritis/Reflux Disease, Gallstone Disease, Familial Pancreatic Cancer, Chronic Pancreatitis, Acute Pancreatitis, Isolated LFT Derangement and Non-specific Abdominal Pain. We also added an additional set of healthy control samples collected from a nested study done in UKCTOCS samples used in a previous paper [62]. The controls matched by age to the PDAC cases in the UKCTOCS cohort that had a time to diagnosis below up to one year were selected. The allocation of these controls to the discovery or held-out validation sets was done according to the division used in our previous work [62]. The number of controls and cases collected for this study can be visualized in Fig 1. UKCTOCS controls are identified as ‘Healthy’.

The discovery held-out validation final split, i.e., with ADEPTS and UKCTOCS samples, put the prevalence of PDAC in the discovery set at close to 8%. The prevalence of PDAC in the resulting validation was approximately 14%. The held-out validation set was isolated and not used in any stage of model and biomarker signature development. This is akin to having a blinded dataset.

Receiver operating characteristic (ROC) curves were constructed for each model to assess diagnostic performance. The area under the curve (AUC) for the ROC curves was used as the metric. ROC curves were generated with the *pROC* R package (version 1.18.0, https://cran.r-project.org/web/packages/pROC/index.html). 95% CI for AUCs were determined by stratified bootstrapping. All AUC confidence intervals crossing 0.5 were considered to be non-significant. P values comparing ROC curves were also calculated using the *pROC* package, under a one-sided bootstrap approach with 10000 runs.

In order to evaluate the association between each of the single markers available for this work, including clinical covariates (see Fig 1 and Table 1), and PDAC status, we created univariate models using a logistic regression model implemented in the *logistf* R package (https://cran.r-project.org/web/packages/logistf/index.html, version 1.24.1). This approach fits a logistic regression model using Firth’s bias reduction method. The reported confidence intervals for odds ratios and tests were based on the profile penalized log likelihood and incorporate the ability to perform tests where contingency tables are asymmetric or contain zeros. The performance of single marker models was also verified in the discovery and held-out validation sets (see Fig B and Tables C and D in S1 Appendix). The same package was also used to verify the association of the presence of symptoms and PDAC status (see Fig 4).

A comprehensive multi-dimensional examination of the collated data was conducted by employing two distinct analytical frameworks. The first was a stacked ensemble algorithm where base-learners were developed according to the same algorithm but in subsets of the discovery set where samples belonging to a specific control diagnosis class were contrasted against the same 24 PDAC cases (see the proportions in Fig 1). The resulting base-learners were then stacked by a logistic regression model, (see Table E for the resulting coefficients and Fig A for the stacking procedures, in S1 Appendix). This approach aimed to leverage the predictive power of multiple models, thereby enhancing the robustness and potentially leading to more precise predictive outcomes [37,43,54]. For each base-learner classifier we resorted to a Recursive Feature Elimination (RFE) routine with logistic regression as the fitting algorithm available through *caret* (version 6.0–93, https://cran.r-project.org/web/packages/caret/index.html). Due to the prevalence of PDAC cases in the whole dataset being low, random under sampling of the majority class, here benign and healthy controls, if the PDACs are pitted against the whole set of controls, would pose a challenge for most algorithms. Therefore, creating an ensemble of classifiers specialised in contrasting a specific diagnostic class against PDAC allowed us more balanced subsets leading to increased performance (Fig 2). For the samples collected from UKCTOCS no symptoms information was available and, therefore, we created a separate classifier associated with this subset of individuals.

Selection of type and number of base-learners has been studied before in other areas [37,43]. Different approaches to this problem have been put forward that either focus on a greedy search for the best ensemble, or rely on diversity based metrics to ensure robustness in external datasets [43]. Here, we have chosen to enforce the diagnosis-specific design to ensure that we relied on clinically relevant features and respected the underlying question of ADEPTS [44]. Nevertheless, we also tested ensemble selection by recursively eliminating base-learners and the best ensemble performer was always the full set of 16 reported above, each contrasting the same PDACs against diagnosis-specific controls.

The second analytical framework followed a more traditional application of state-of-the-art algorithms to the whole discovery set. We tested 3 different algorithms under this framework: random forests (RRF, version 1.9.4, https://cran.r-project.org/web/packages/RRF/index.html); extreme gradient boosting trees (xgbTree, version 1.6.0.1, https://cran.r-project.org/web/packages/xgboost/index.html); and a generalized linear model with RFE (RFE glm). It is important to clearly stress the differences between this RFE glm model and the stacked ensemble model reported above. The additional RFE glm model, despite using similar techniques to each base-learner in the ensemble approach, was applied to the whole discovery data set, without division of the control samples into diagnosis classes. This resulted in one model only as opposed to 16, and therefore there is no need for stacking under this additional approach.

All ensemble base-models as well as all the additional state-of-the-art algorithms mentioned above, i.e., xgbTree, RRF and RFE glm, were trained in the discovery subset with leave-one-out cross validation in order to find the optimal set of input features or the optimum hyperparameters (see Table M in S1 Appendix). 1000 random parameter combinations were tested to achieved optimum performance.

We tested 3 subsampling algorithms combined with each of the models: oversampling of the minority class, the Synthetic Minority Oversampling Technique (SMOTE) [41,63] algorithm and under sampling of the majority class (see Table F in S1 Appendix). The sub-sampling routines were performed within the cross-validation procedure to avoid overfitting [64–66].

The RFE associated with 2 of the algorithms mentioned above was also performed within the cross-validation folds. This reduces data leakage and overfitting due to the fact that feature selection is performed for each training fold and a rank of potential feature groups is created based on their cross-validation performance [64–67], thus leading to the most robust option.

To verify if the PDAC index developed with the ensemble stacked approach had any association with metrics used in the clinic but not taken into account in any stage of algorithm training, we also gathered the QCancer pancreatic score [21] for individuals in the ADEPTS study (see Fig 6). This allowed us further validation of the diagnosis-based ensemble index and a view of its potential as a complementary measure.

The procedure for assessing feature importance in each base learner was a model-agnostic method based on a simple feature importance ranking measure [68], implemented in the R package *vip* (version 0.3.2, https://cran.r-project.org/web/packages/vip/index.html). The model-agnostic interpretability, by decoupling the interpretation from the model itself, introduces a level of flexibility that enables its application across any supervised learning algorithm. Despite the algorithm used for each diagnosis-specific classifier being the same, the model-agnostic approach allows us to be able to generalise the computed importances to other work in the literature.

Enrichment analysis for each of the signatures developed was performed with the *gprofiler2* R package (version 0.2.1, https://cran.r-project.org/web/packages/gprofiler2/index.html). A threshold for multiple comparison correction under the framework of false discovery rate was instituted at 0.05.

## Supporting information

S1 Appendix Supplementary Figures, Tables and Text. All supplementary figures and tables cited in the main text as well as supplementary text on the application of a reduced, 8-marker signature as a differentiator of PDAC from healthy and benign controls.
(DOCX)

S1 Data Fig 3 scaled importance data. The data for the scaled importances for each diagnosis class. See Materials and methods section for details.
(XLSX)

## References

[1] Kamisawa T, Wood LD, Itoi T, Takaori K. Pancreatic cancer. The Lancet. 2016;388:73–85. doi: 10.1016/S0140-6736(16)00141-0 26830752

[2] Sung H, Ferlay J, Siegel RL, Laversanne M, Soerjomataram I, Jemal A, et al. Global Cancer Statistics 2020: GLOBOCAN Estimates of Incidence and Mortality Worldwide for 36 Cancers in 185 Countries. CA: A Cancer Journal for Clinicians. 2021;71:209–49. doi: 10.3322/caac.21660 33538338

[3] Carioli G, Malvezzi M, Bertuccio P, Boffetta P, Levi F, Vecchia CL, et al. European cancer mortality predictions for the year 2021 with focus on pancreatic and female lung cancer. Annals of Oncology. 2021;32:478–87. doi: 10.1016/j.annonc.2021.01.006 33626377

[4] Marchegiani G, Andrianello S, Malleo G, De Gregorio L, Scarpa A, Mino-Kenudson M, et al. Does Size Matter in Pancreatic Cancer?: Reappraisal of Tumour Dimension as a Predictor of Outcome Beyond the TNM. Annals of Surgery. 2017;266(1). doi: 10.1097/SLA.0000000000001837 27322188

[5] Zerboni G, Signoretti M, Crippa S, Falconi M, Arcidiacono PG, Capurso G. Systematic review and meta-analysis: Prevalence of incidentally detected pancreatic cystic lesions in asymptomatic individuals. Pancreatology. 2019;19:2–9. doi: 10.1016/j.pan.2018.11.014 30503370

[6] Pereira SP, Oldfield L, Ney A, Hart PA, Keane MG, Pandol SJ, et al. Early detection of pancreatic cancer. The Lancet Gastroenterology and Hepatology. 2020. doi: 10.1016/S2468-1253(19)30416-9 32135127 PMC7380506

[7] Aslanian HR, Lee JH, Canto MI. AGA Clinical Practice Update on Pancreas Cancer Screening in High-Risk Individuals: Expert Review. Gastroenterology. 2020;159:358–62. doi: 10.1053/j.gastro.2020.03.088 32416142

[8] Owens DK, Davidson KW, Krist AH, Barry MJ, Cabana M, Caughey AB, et al. Screening for Pancreatic Cancer. JAMA. 2019;322:438–. 31386141 10.1001/jama.2019.10232

[9] Chhoda A, Vodusek Z, Wattamwar K, Mukherjee E, Gunderson C, Grimshaw A, et al. Late-Stage Pancreatic Cancer Detected During High-Risk Individual Surveillance: A Systematic Review and Meta-Analysis. Gastroenterology. 2022;162:786–98. doi: 10.1053/j.gastro.2021.11.021 34813861

[10] European evidence-based guidelines on pancreatic cystic neoplasms. Gut. 2018;67:789–804. doi: 10.1136/gutjnl-2018-316027 29574408 PMC5890653

[11] Walter FM, Mills K, Mendonça SC, Abel GA, Basu B, Carroll N, et al. Symptoms and patient factors associated with diagnostic intervals for pancreatic cancer (SYMPTOM pancreatic study): a prospective cohort study. The Lancet Gastroenterology & Hepatology. 2016;1:298–306. doi: 10.1016/S2468-1253(16)30079-6 28404200 PMC6358142

[12] Lukacs G, Kovacs A, Csanadi M, Moizs M, Repa I, Kalo Z, et al. Benefits Of Timely Care In Pancreatic Cancer: A Systematic Review To Navigate Through The Contradictory Evidence. Cancer Manag Res. 2019;11:9849–61. doi: 10.2147/CMAR.S221427 31819622 PMC6875504

[13] Yu J, Blackford AL, Dal Molin M, Wolfgang CL, Goggins M. Time to progression of pancreatic ductal adenocarcinoma from low-to-high tumour stages. Gut. 2015;64(11):1783–9. doi: 10.1136/gutjnl-2014-308653 25636698 PMC4520782

[14] Ahn SJ, Choi SJ, Kim HS. Time to Progression of Pancreatic Cancer: Evaluation with Multi-Detector Computed Tomography. J Gastrointest Cancer. 2017;48(2):164–9. doi: 10.1007/s12029-016-9876-7 27699624

[15] Lyratzopoulos G, Wardle J, Rubin G. Rethinking diagnostic delay in cancer: how difficult is the diagnosis? BMJ. 2014;349:g7400. doi: 10.1136/bmj.g7400 25491791

[16] Escorza-Calzada S, Rosas-Camargo V, Melchor-Ruan J, Meneses-Medina M, Cedro-Tanda A, Huitzil-Melendez F. P-319 Delay in pancreatic cancer diagnosis and treatment: Call to action. Annals of Oncology. 2023;34:S127.

[17] Keane MG, Horsfall L, Rait G, Pereira SP. A case-control study comparing the incidence of early symptoms in pancreatic and biliary tract cancer. BMJ open. 2014;4:e005720–e. doi: 10.1136/bmjopen-2014-005720 25410605 PMC4244441

[18] Liao W, Clift AK, Patone M, Coupland C, González-Izquierdo A, Pereira SP, et al. Identifying symptoms associated with diagnosis of pancreatic exocrine and neuroendocrine neoplasms: a nested case-control study of the UK primary care population. British Journal of General Practice. 2021;71:e836–e45. doi: 10.3399/BJGP.2021.0153 34544691 PMC8463137

[19] Schmidt-Hansen M, Berendse S, Hamilton W. Symptoms of Pancreatic Cancer in Primary Care. Pancreas. 2016;45:814–8. 26495795 10.1097/MPA.0000000000000527

[20] Mizrahi JD, Surana R, Valle JW, Shroff RT. Pancreatic cancer. The Lancet. 2020;395:2008–20. 10.1016/S0140-6736(20)30974-0 32593337

[21] Hippisley-Cox J, Coupland C. Development and validation of risk prediction algorithms to estimate future risk of common cancers in men and women: prospective cohort study. BMJ Open. 2015;5:e007825–e. doi: 10.1136/bmjopen-2015-007825 25783428 PMC4368998

[22] Hamilton W. The CAPER studies: five case-control studies aimed at identifying and quantifying the risk of cancer in symptomatic primary care patients. British Journal of Cancer. 2009;101:S80–S6. doi: 10.1038/sj.bjc.6605396 19956169 PMC2790706

[23] Usher-Smith J, Emery J, Hamilton W, Griffin SJ, Walter FM. Risk prediction tools for cancer in primary care. British Journal of Cancer. 2015;113:1645–50. doi: 10.1038/bjc.2015.409 26633558 PMC4701999

[24] Price S, Spencer A, Medina-Lara A, Hamilton W. Availability and use of cancer decision-support tools: a cross-sectional survey of UK primary care. Br J Gen Pract. 2019;69(684):e437–e43. doi: 10.3399/bjgp19X703745 31064743 PMC6592323

[25] Humphris JL, Chang DK, Johns AL, Scarlett CJ, Pajic M, Jones MD, et al. The prognostic and predictive value of serum CA19.9 in pancreatic cancer. Annals of Oncology. 2012;23:1713–22. doi: 10.1093/annonc/mdr561 22241899 PMC3387824

[26] Luo G, Jin K, Deng S, Cheng H, Fan Z, Gong Y, et al. Roles of CA19-9 in pancreatic cancer: Biomarker, predictor and promoter. Biochimica et Biophysica Acta (BBA)—Reviews on Cancer. 2021;1875(2):188409. doi: 10.1016/j.bbcan.2020.188409 32827580

[27] Ballehaninna UK, Chamberlain RS. The clinical utility of serum CA 19–9 in the diagnosis, prognosis and management of pancreatic adenocarcinoma: An evidence based appraisal. J Gastrointest Oncol. 2012;3(2):105–19. doi: 10.3978/j.issn.2078-6891.2011.021 22811878 PMC3397644

[28] Kim Y, Yeo I, Huh I, Kim J, Han D, Jang JY, et al. Development and Multiple Validation of the Protein Multi-marker Panel for Diagnosis of Pancreatic Cancer. Clin Cancer Res. 2021;27(8):2236–45. doi: 10.1158/1078-0432.CCR-20-3929 33504556

[29] Potjer TP. Pancreatic cancer surveillance and its ongoing challenges: is it time to refine our eligibility criteria? Gut. 2022;71:1047–9. doi: 10.1136/gutjnl-2021-324739 34145046

[30] Boyd LNC, Ali M, Leeflang MMG, Treglia G, de Vries R, Le Large TYS, et al. Diagnostic accuracy and added value of blood-based protein biomarkers for pancreatic cancer: A meta-analysis of aggregate and individual participant data. EClinicalMedicine. 2023;55:101747. doi: 10.1016/j.eclinm.2022.101747 36457649 PMC9706531

[31] Kane LE, Mellotte GS, Mylod E, O’Brien RM, O’Connell F, Buckley CE, et al. Diagnostic Accuracy of Blood-based Biomarkers for Pancreatic Cancer: A Systematic Review and Meta-analysis. Cancer Res Commun. 2022;2(10):1229–43. doi: 10.1158/2767-9764.CRC-22-0190 36969742 PMC10035398

[32] Cohen JD, Li L, Wang Y, Thoburn C, Afsari B, Danilova L, et al. Detection and localization of surgically resectable cancers with a multi-analyte blood test. Science. 2018;359:926–30. doi: 10.1126/science.aar3247 29348365 PMC6080308

[33] Liu MC, Oxnard GR, Klein EA, Swanton C, Seiden MV, Liu MC, et al. Sensitive and specific multi-cancer detection and localization using methylation signatures in cell-free DNA. Annals of Oncology. 2020;31(6):745–59. doi: 10.1016/j.annonc.2020.02.011 33506766 PMC8274402

[34] Klein EA, Richards D, Cohn A, Tummala M, Lapham R, Cosgrove D, et al. Clinical validation of a targeted methylation-based multi-cancer early detection test using an independent validation set. Annals of Oncology. 2021;32(9):1167–77. doi: 10.1016/j.annonc.2021.05.806 34176681

[35] Neal RD, Johnson P, Clarke CA, Hamilton SA, Zhang N, Kumar H, et al. Cell-Free DNA-Based Multi-Cancer Early Detection Test in an Asymptomatic Screening Population (NHS-Galleri): Design of a Pragmatic, Prospective Randomised Controlled Trial. Cancers (Basel). 2022;14(19). 10.3390/cancers14194818 PMC9564213 36230741

[36] Menon U, Gentry-Maharaj A, Burnell M, Singh N, Ryan A, Karpinskyj C, et al. Ovarian cancer population screening and mortality after long-term follow-up in the UK Collaborative Trial of Ovarian Cancer Screening (UKCTOCS): a randomised controlled trial. Lancet. 2021;397(10290):2182–93. doi: 10.1016/S0140-6736(21)00731-5 33991479 PMC8192829

[37] Nené NR, Ney A, Nazarenko T, Blyuss O, Johnston HE, Whitwell HJ, et al. Serum biomarker-based early detection of pancreatic ductal adenocarcinomas with ensemble learning. Communications Medicine. 2023;3:10–. doi: 10.1038/s43856-023-00237-5 36670203 PMC9860022

[38] James AD, Richardson DA, Oh IW, Sritangos P, Attard T, Barrett L, et al. Cutting off the fuel supply to calcium pumps in pancreatic cancer cells: role of pyruvate kinase-M2 (PKM2). Br J Cancer. 2020;122(2):266–78. doi: 10.1038/s41416-019-0675-3 31819190 PMC7052184

[39] Matull WR, Andreola F, Loh A, Adiguzel Z, Deheragoda M, Qureshi U, et al. MUC4 and MUC5AC are highly specific tumour-associated mucins in biliary tract cancer. Br J Cancer. 2008;98(10):1675–81. doi: 10.1038/sj.bjc.6604364 18475301 PMC2391120

[40] Cuenco J, Wehnert N, Blyuss O, Kazarian A, Whitwell HJ, Menon U, et al. Identification of a serum biomarker panel for the differential diagnosis of cholangiocarcinoma and primary sclerosing cholangitis. Oncotarget. 2018;9(25):17430–42. doi: 10.18632/oncotarget.24732 29707118 PMC5915126

[41] Blagus R, Lusa L. SMOTE for high-dimensional class-imbalanced data. BMC Bioinformatics. 2013;14:106. doi: 10.1186/1471-2105-14-106 23522326 PMC3648438

[42] He H, Bai Y, Garcia EA, Li S. ADASYN: Adaptive synthetic sampling approach for imbalanced learning. 2008 IEEE International Joint Conference on Neural Networks (IEEE World Congress on Computational Intelligence) SP—1322. 2008.

[43] Whalen S, Pandey G, editors. A Comparative Analysis of Ensemble Classifiers: Case Studies in Genomics. 2013 IEEE 13th International Conference on Data Mining; 2013 7–10 Dec. 2013.

[44] Pereira S, Hippisley-Cox J, Timms J, Hsuan J, Fusai K, Williams N, et al. ADEPTS (Accelerated Diagnosis of neuroEndocrine and Pancreatic TumourS) and EDRA (Early Diagnosis Research Alliance). Pancreatology. 2020;20(8):e14.

[45] Bestari MB, Joewono IR, Syam AF. A Quest for Survival: A Review of the Early Biomarkers of Pancreatic Cancer and the Most Effective Approaches at Present. Biomolecules. 2024;14(3). doi: 10.3390/biom14030364 38540782 PMC10968439

[46] Ansari D, Toren W, Zhou Q, Hu D, Andersson R. Proteomic and genomic profiling of pancreatic cancer. Cell Biol Toxicol. 2019;35(4):333–43. doi: 10.1007/s10565-019-09465-9 30771135 PMC6757097

[47] Root A, Allen P, Tempst P, Yu K. Protein Biomarkers for Early Detection of Pancreatic Ductal Adenocarcinoma: Progress and Challenges. Cancers (Basel). 2018;10(3). 10.3390/cancers10030067 PMC5876642 29518918

[48] O’Brien DP, Sandanayake NS, Jenkinson C, Gentry-Maharaj A, Apostolidou S, Fourkala EO, et al. Serum CA19-9 is significantly up-regulated up to 2 years prior to diagnosis with pancreatic cancer: implications for early disease detection. Clin Cancer Res. 2015;21(3):622–31. 24938522 10.1158/1078-0432.CCR-14-0365 PMC4181906

[49] QCancer-2018 risk calculator for men: http://qcancer.org/male [

[50] QCancer-2018 risk calculator for women: http://qcancer.org/female [

[51] [Available from: https://www.nice.org.uk.

[52] Sturm N, Ettrich TJ, Perkhofer L. The Impact of Biomarkers in Pancreatic Ductal Adenocarcinoma on Diagnosis, Surveillance and Therapy. Cancers. 2022;14:217–. doi: 10.3390/cancers14010217 35008381 PMC8750069

[53] Azizian A, Ruhlmann F, Krause T, Bernhardt M, Jo P, Konig A, et al. CA19-9 for detecting recurrence of pancreatic cancer. Sci Rep. 2020;10(1):1332. doi: 10.1038/s41598-020-57930-x 31992753 PMC6987233

[54] Caruana R, Niculescu-Mizil A, Crew G, Ksikes A. Ensemble selection from libraries of models. Proceedings of the twenty-first international conference on Machine learning; Banff, Alberta, Canada: Association for Computing Machinery; 2004. p. 18.

[55] Sagi O, Rokach L. Ensemble learning: A survey. WIREs Data Mining and Knowledge Discovery. 2018;8(4):e1249.

[56] Benjamin JL, Maruan A-S, Amir A, Jennifer W, Sami L, Eric PX. Discriminative Subtyping of Lung Cancers from Histopathology Images via Contextual Deep Learning. medRxiv. 2022:2020.06.25.20140053.

[57] Placido D, Yuan B, Hjaltelin JX, Zheng C, Haue AD, Chmura PJ, et al. A deep learning algorithm to predict risk of pancreatic cancer from disease trajectories. Nat Med. 2023;29(5):1113–22. doi: 10.1038/s41591-023-02332-5 37156936 PMC10202814

[58] Whitwell HJ, Worthington J, Blyuss O, Gentry-Maharaj A, Ryan A, Gunu R, et al. Improved early detection of ovarian cancer using longitudinal multimarker models. British Journal of Cancer. 2020;122(6):847–56. doi: 10.1038/s41416-019-0718-9 31937926 PMC7078315

[59] Menon U, Gentry-Maharaj A, Hallett R, Ryan A, Burnell M, Sharma A, et al. Sensitivity and specificity of multimodal and ultrasound screening for ovarian cancer, and stage distribution of detected cancers: results of the prevalence screen of the UK Collaborative Trial of Ovarian Cancer Screening (UKCTOCS). Lancet Oncol. 2009;10(4):327–40. doi: 10.1016/S1470-2045(09)70026-9 19282241

[60] Menon U, Gentry-Maharaj A, Ryan A, Sharma A, Burnell M, Hallett R, et al. Recruitment to multicentre trials—lessons from UKCTOCS: descriptive study. Bmj. 2008;337:a2079. doi: 10.1136/bmj.a2079 19008269 PMC2583394

[61] Data normalization and standardization [Available from: https://www.olink.com/content/uploads/2021/09/olink-data-normalization-white-paper-v2.0.pdf.

[62] Nené NR, Ney A, Nazarenko T, Blyuss O, Johnston HE, Whitwell HJ, et al. Early detection of pancreatic ductal adenocarcinomas with an ensemble learning model based on a panel of protein serum biomarkers. medRxiv. 2021:2021.12.02.21267187.

[63] Nitesh V. C, Bowyer KW, Hall LO, Kegelmeyer WP. SMOTE: synthetic minority over-sampling technique. Journal of Artificial Intelligence Research. 2002;16(1): 321–57.

[64] Ambroise C, McLachlan GJ. Selection bias in gene extraction on the basis of microarray gene-expression data. Proc Natl Acad Sci U S A. 2002;99(10):6562–6. doi: 10.1073/pnas.102102699 11983868 PMC124442

[65] Cawley GC, Talbot NL. On over-fitting in model selection and subsequent selection bias in performance evaluation. The Journal of Machine Learning Research. 2010;11:2079–107.

[66] Teschendorff AE. Avoiding common pitfalls in machine learning omic data science. Nat Mater. 2019;18(5):422–7. doi: 10.1038/s41563-018-0241-z 30478452

[67] Whalen S, Schreiber J, Noble WS, Pollard KS. Navigating the pitfalls of applying machine learning in genomics. Nat Rev Genet. 2022;23(3):169–81. doi: 10.1038/s41576-021-00434-9 34837041

[68] Scholbeck CA, Molnar C, Heumann C, Bischl B, Casalicchio G, editors. Sampling, Intervention, Prediction, Aggregation: A Generalized Framework for Model-Agnostic Interpretations. Machine Learning and Knowledge Discovery in Databases; 2020 2020//; Cham: Springer International Publishing.

