# Supplementary material for: Identification of a serum proteomic biomarker panel using diagnosis specific ensemble learning and symptoms for early pancreatic cancer detection
Source: PLoS Comput Biol. 2024 Aug 29;20(8):e1012408. doi: 10.1371/journal.pcbi.1012408 (PMC11389906; doi:10.1371/journal.pcbi.1012408)
Supplement: S1 Appendix — All supplementary figures and tables cited in the main text as well as supplementary text on the application of a reduced, 8-marker signature as a differentiator of PDAC from healthy and benign controls. (DOCX) [file pcbi.1012408.s001.docx]

Supporting Information

Title: Identification of a serum proteomic biomarker panel using diagnosis specific ensemble learning and symptoms for early pancreatic cancer detection

**Authors:**

Alexander Ney^1,^¶, Nuno R. Nené^2,3,4,^¶, Eva Sedlak^2^, Pilar Acedo^1^, Oleg Blyuss^5,6^, Harry J. Whitwell^2,7,8^, Eithne Costello^9^, Aleksandra Gentry-Maharaj^2,10^, Norman R. Williams^11^, Usha Menon^10^, Giuseppe K. Fusai^12^, Alexey Zaikin^2,13,14,15,16^, Stephen P. Pereira^1^.

**Affiliations:**

^1^ Institute for Liver and Digestive Health, University College London, London, United Kingdom

^2^ Department of Women’s Cancer, EGA Institute for Women’s Health, University College London, London, United Kingdom

^3^ Cancer Institute, University College London, London, United Kingdom.

^4^ Department of Statistical Science, University College London, London, United Kingdom

^5^ Center for Cancer Prevention, Detection and Early Diagnosis, Wolfson Institute of Population Health, Queen Mary University of London, London, United Kingdom

^6^ Department of Pediatrics and Pediatric Infectious Diseases, Institute of Child´s Health, Sechenov First Moscow State Medical University (Sechenov University), Moscow, Russia

^7^ National Phenome Centre and Imperial Clinical Phenotyping Centre, Department of Metabolism, Digestion and Reproduction, IRDB, Building Imperial College London, London, United Kingdom

^8^ Section of Bioanalytical Chemistry, Division of Systems Medicine, Department of Metabolism, Digestion and Reproduction, Sir Alexander Fleming Building, Imperial College London, London, United Kingdom

^9^ Department of Molecular and Clinical Cancer Medicine, University of Liverpool, Liverpool, United Kingdom

^10^ MRC Clinical Trials Unit at UCL, Institute of Clinical Trials and Methodology, University College London, London, United Kingdom

^11^ Division of Surgery & Interventional Science, University College London, London, United Kingdom

^12^ HPB & Liver Transplant Unit, Royal Free London, London, United Kingdom

^13^ Institute for Cognitive Neuroscience, University Higher School of Economics, Moscow, Russia

^14^ Department of Mathematics, University College London, London, United Kingdom

^15^ Centre for Cognition and Decision making, Institute for Cognitive Neuroscience, HSE University, Moscow, Russia

^16^ Life Improvement by Future Technologies (LIFT) Center, Skolkovo, Moscow, Russia

¶ These authors contributed equally to the work

*Email: alexney@me.com; [nuno.nene.10@ucl.ac.uk](mailto:nuno.nene.10@ucl.ac.uk); [stephen.pereira@ucl.ac.uk](mailto:stephen.pereira@ucl.ac.uk)

In this supporting information we provide the additional tables and figures cited in the main text. We also described the results with a reduced biomarker signature based on the diagnosis-based ensemble approach.

**Supplementary Text**

**Application of a reduced, 8-marker signature as a differentiator of PDAC from healthy and benign controls**

Across all conditions, 8 features with relatively higher scaled importance that differentiated controls from PDAC patients were selected (Fig 3, main text). Importance was measured by the contribution of a specific feature to the output of the model (see Methods, main text), in our case the probability of PDAC. The set of 8 features included CA19-9, VWF, CPE, CTSV, CEACAM1 and CD160 together with Diabetes and Age as clinicodemographic variables. Diabetes was a predictor of the differences between PDAC against familial cases, gastric reflux disease (GORD), sphincter of oddi (SOD) dysfunction, as well as healthy controls.

CA19-9 levels were only selected as a top discriminating feature against PDACs in patients with suspected sphincter of Oddi dysfunction, benign liver disease, irritable bowel syndrome (IBS), those with isolated LFT derangements as well as distinguished healthy subjects and those with other cancers (Fig 3, in main text, and Table N, in S1 Appendix), from PDAC patients.

Von Willebrand Factor (VWF) levels differentiated PDAC from symptomatic patients with pancreatic cysts, benign biliary duct diseases, non-abdominal conditions, patients with family history of PDAC, those with GORD as well as healthy subjects.

The immunoglobulin like surface antigen molecule CD160 (peripheral natural killer cells and CD8^+^ T lymphocytes) (1) and a proposed immune checkpoint inhibitor, was selected as a significant differentiator of PDAC from benign biliary tract diseases (IgG4 disease), SOD dysfunction, IBS as well as in familial pancreatic cancer subjects and other cancers. Cathepsin V (CTSV) levels were also a predictor of multiple conditions against PDAC, including benign biliary diseases and in subjects belonging to the familial PC cohort. In healthy subjects, however, this feature did not show significant importance as a differentiator from PDAC.

Serum levels of the metallo-carboxypeptidase E (CPE) were a feature selected as significant in five conditions (acute pancreatitis, gallstones and IgG4 disease, SOD dysfunction and GORD) as well as a differentiator in those with FH of PC. A higher scaled importance was attributed to this enzyme against CA19-9 when differentiating acute and chronic pancreatitis (CP), isolated LFT derangements, unexplained abdominal pain and non-abdominal conditions versus PDAC (Fig 3 in main text).

THE CEA cell adhesion molecule (CEACAM1) was selected as a feature in patients with non-explained recurrent abdominal pain, isolated LFT derangements, GORD, SOD dysfunction as well as a feature selected against non-pancreatic cancers.

Chronic pancreatitis (CP) is a known risk factor for PDAC. In our index signature, the protein markers selected against PDAC included ESM1, ICOSLG, CTSV, CXL17 (CXC motif chemokine ligand 17), IL6ST, ITGAV, GZMB (granzyme B; secreted serine protease), with a reduced risk for cancer in Caucasian ethnicity (2-4).

We therefore opted to assess their combined performance against CA19-9 as a single marker and the full ensemble described in the main text. Using a similar stacking procedure as before, a reduced model was trained using the same ensemble approach as that highlighted in the main text but with only 8 features as the input. The reduced signature predicted PDAC still with a high AUC value of 0.97 (95% CI 0.95-0.98), sensitivity 0.98 (95% CI 0.95-1), PPV 0.92 (95% CI 0.91-0.92) and NPV of 0.98 (95% CI 0.94-1) at 90% specificity, in the discovery set (Fig EC in S1 Appendix). In the held-out validation set, however, the performance of the 8-marker signature was significantly reduced (p= 0.00038, one-sided) compared to the full stacked model (AUC of 0.84 (95% CI 0.75-0.94), sensitivity 0.64 (95% CI 0.36-0.82), PPV 0.47 (95% CI 0.33-0.53) yet with a NPV of 0.95 (95% CI 0.91-0.97) at 90% specificity (Fig ED in S1 Appendix), and only marginally superior to CA19-9 as a single marker (p=0.18, one-sided). On the other hand, the 8-marker signature still outperformed CA19-9 by a relatively large margin when predicting PDAC against healthy UKCTOCS controls in the validation set: AUC_redsig_ of 0.93 (95% CI 0.84 - 1), sensitivity_redsig_ of 0.86 (95% CI 0.54 - 1), PPV_redsig_ 0.94 (95% CI 0.90 – 0.94), NPV_redsig_ 0.80 (95% CI 0.54 – 1); AUC_CA19-9_ of 0.84 (95% CI 0.70 - 0.97), sensitivity _CA19-9_ of 0.68 (95% CI 0.5 – 0.91), PPV _CA19-9_ 0.92 (95% CI 0.89 – 0.94), NPV _CA19-9_  0.62 (95% CI 0.52 – 0.85), at 90% specificity. Under a bootstrap test this AUC difference is significant p= 0.025 (one-sided). In addition, it also outperformed the full PDAC ensemble model when predicting PDAC against healthy controls in the validation set, although the differences were not significant (p=0.2, one-sided): AUC_sig_ of 0.90 (95% CI 0.77 – 1), sensitivity_sig_ of 0.86 (95% CI 0.54 – 1), PPV_sig_ 0.94 (95% CI 0.92 – 0.94), NPV_sig_ 0.80 (95% CI 0.66 – 1) at 90% specificity.

If on the other hand the reduced signature is validated in ADEPTS samples only, i.e. in PDACs plus benign disease controls, the performance of the reduced signature is far inferior to the full signature: AUC_redsig_ of 0.83 (95% CI 0.73 – 0.93) (p= 0.0009 when compared with AUC_sig_, one-sided test), sensitivity_redsig_ 0.59 (95% CI 0.27 – 0.82), PPV_redsig_ 0.47 (95% CI 0.29 – 0.55) and NPV_redsig_ 0.94 (95% CI 0.89 – 0.97), at 90% specificity. This further justifies the use of the full ensemble signature in blind data sets and in a scenario where there is limited information on a patient trajectory, despite its increased complexity.

In consideration of the marker importance in the reduced model, no marker received a null significance across all diagnostic specific base-learners, a divergence from observations in the comprehensive signature. CA19-9 emerged with the largest average importance across conditions, which also contrasted with the full model. Age and VWF were ranked with elevated average significance across diverse conditions. It's pertinent to note that CPE levels manifested diminished scaled importance in discerning healthy controls from PDAC, particularly when juxtaposed against CA19-9, age, and CEACAM1 (refer to Fig F in S1 Appendix).

**Supplementary Figures**

**Fig A. Flow diagram for the diagnosis-specific stack ensemble classifier.** The general linear stack model presented in the main text was built according to this diagram, where base-learners are trained in groups of control samples with a specific diagnosis and the same PDAC cases. The stacking procedure has 2 steps. First, the probability output vectors for each base-learner are concatenated, thus leading to n probability vectors, where n is the number of diagnosis subclasses. Second, these vectors are subsequently used to populate the diagonal blocks of a large probability matrix. The off-diagonal probability blocks are generated by using the base models trained in a specific diagnosis subclass plus the same PDACs, which therefore amounts to computing cross diagnosis predictions. The resulting large matrix has n columns and is then used to train the meta-learner which outputs the final probability vector. For the purposes of applying the resulting trained models to the validation set, the flow of the diagram is the same as before, but the feature matrix will have a different number of samples. See Methods section in the main text for further details.

**Fig B. Biomarker ranks in the discovery set. A** Distribution and ranks of biomarkers by p values in the discovery set. Purple dashed line corresponds to -Log [0.05]. **B** Receiver Operating Curve (ROC) Area Under the Curve (AUC), Sensitivity (Sens), Positive Predictive Value (PPV) and Negative Predictive Value (NPV) at 90% Specificity (Spec) performance of single marker models in the held-out validation set. OR stands for odds-ratio, with dot size proportional to the calculated values. Red and blue OR points represent OR > 1 (favours pancreatic ductal adenocarcinoma (PDAC) case status) and OR < 1 (favours Control status), respectively. p values were calculated according to a logistic regression model with a bias reduction method. Performances were calculated with the single feature models developed in the discovery set. The ROC AUC significance threshold is also represented by a purple dashed line at 0.5. Error bars in figures corresponding to the validation set correspond to 95% Confidence Intervals (CI), calculated by stratified bootstrapping 2000 times. See statistical analysis section in Methods (main text) for further details and Tables C and D (in S1 Appendix).

**
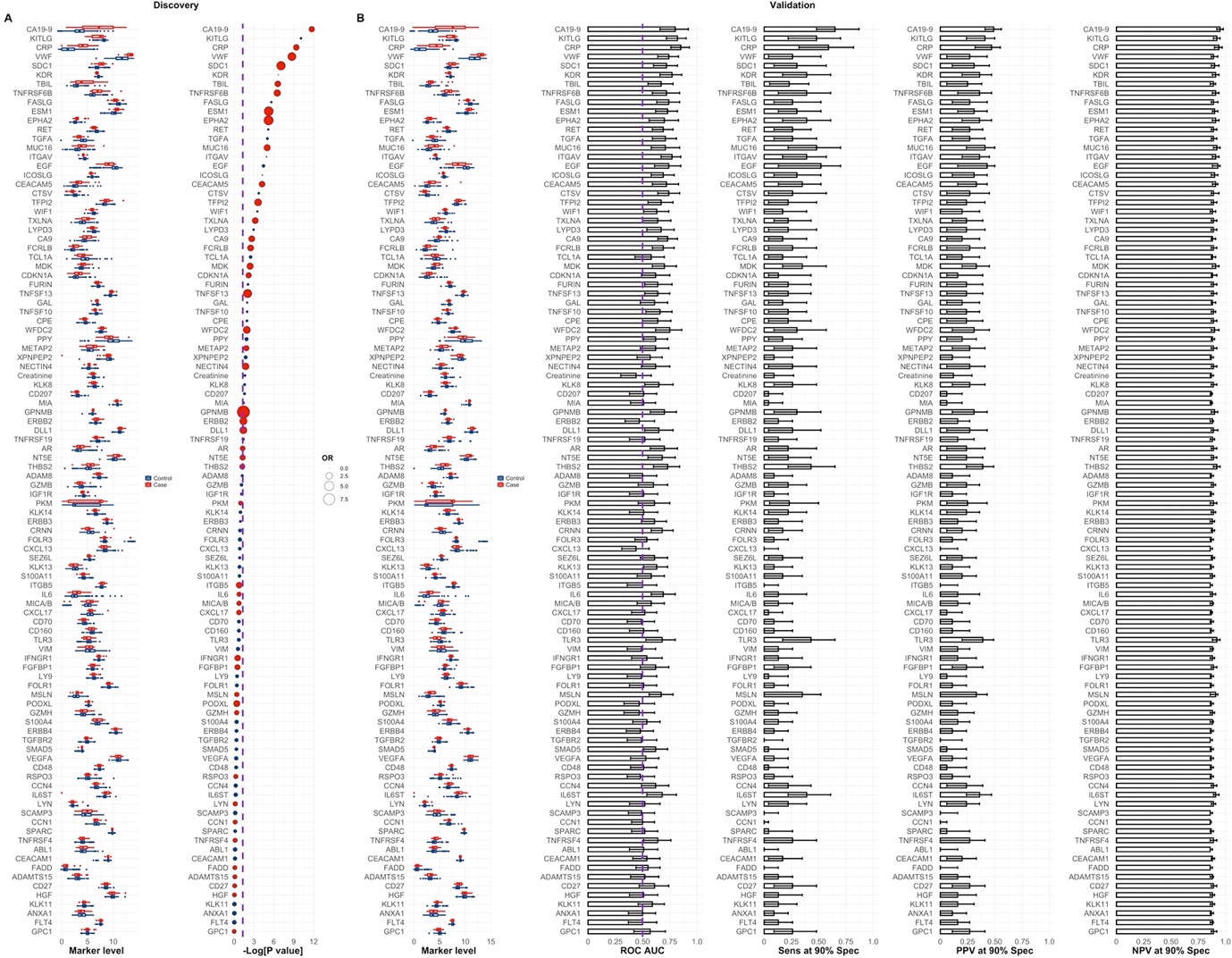
**

**Fig C. Enrichment analysis for the diagnosis-specific ensemble model.** g:Profiler terms for the set of features selected by the full stack ensemble. **A** Kyoto Encyclopaedia of Genes and Genomes (KEGG) pathways. **C** Reactome Pathway Database (REAC). **E** WikiPathways (WP). **G** Gene ontology terms biological process (GO: BP). The respective adjusted p-values associated with each enrichment term or pathway are plotted in **B**, **D**, **F** and **H**. See also Fig 3 (main text). See Statistical Analysis in Methods for further details (main text).

**
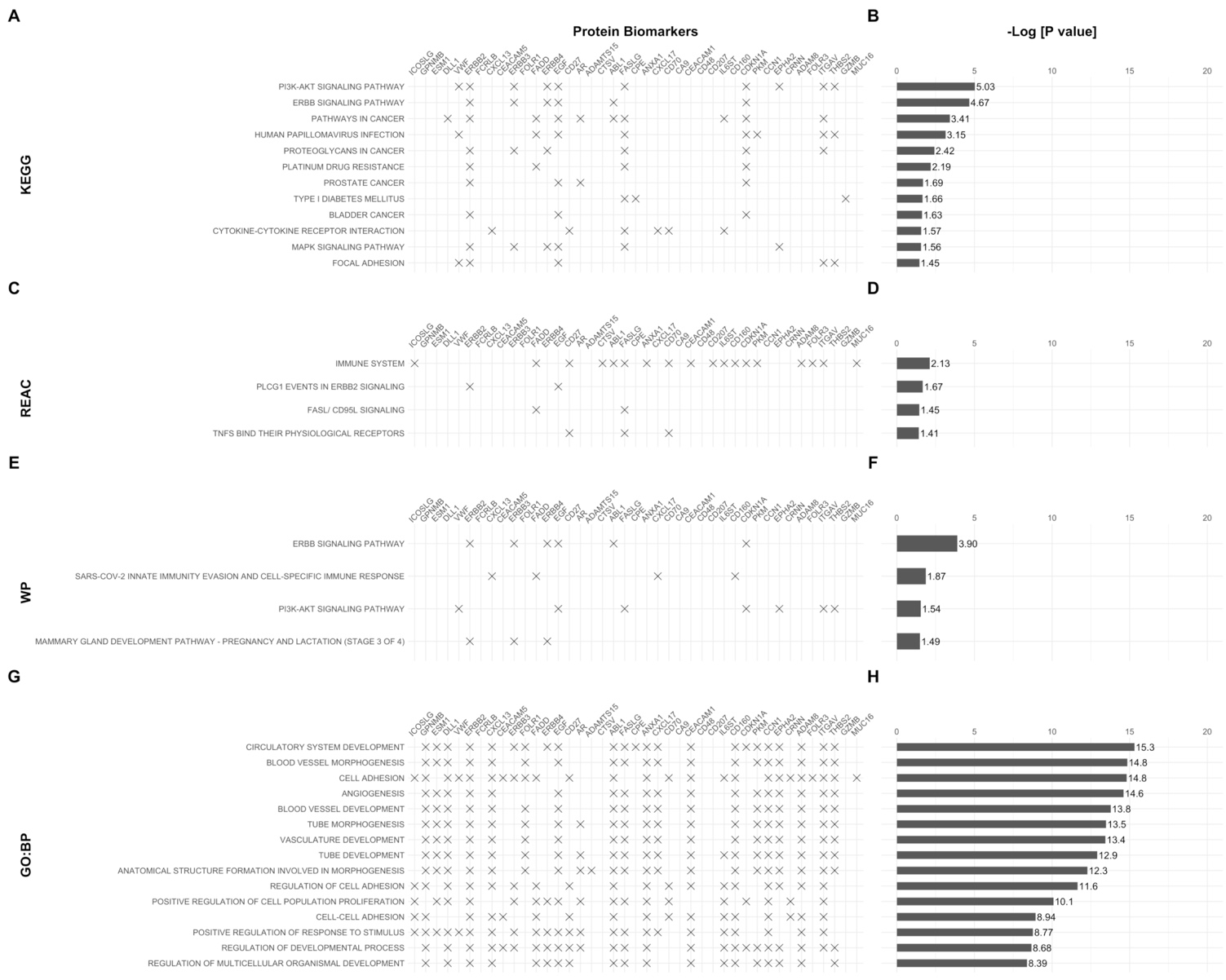
**

**Fig D. Matrix for associated symptoms for each sample.** Each sample in the whole set collected from ADEPTS cohort is represented by the columns. The colour coding corresponds to the diagnosis class associated with each sample. Symptoms are represented in each row. Both columns and rows are clustered according to their symptoms pattern. Black represents presence of sample with the respective symptoms and diagnosis.

**
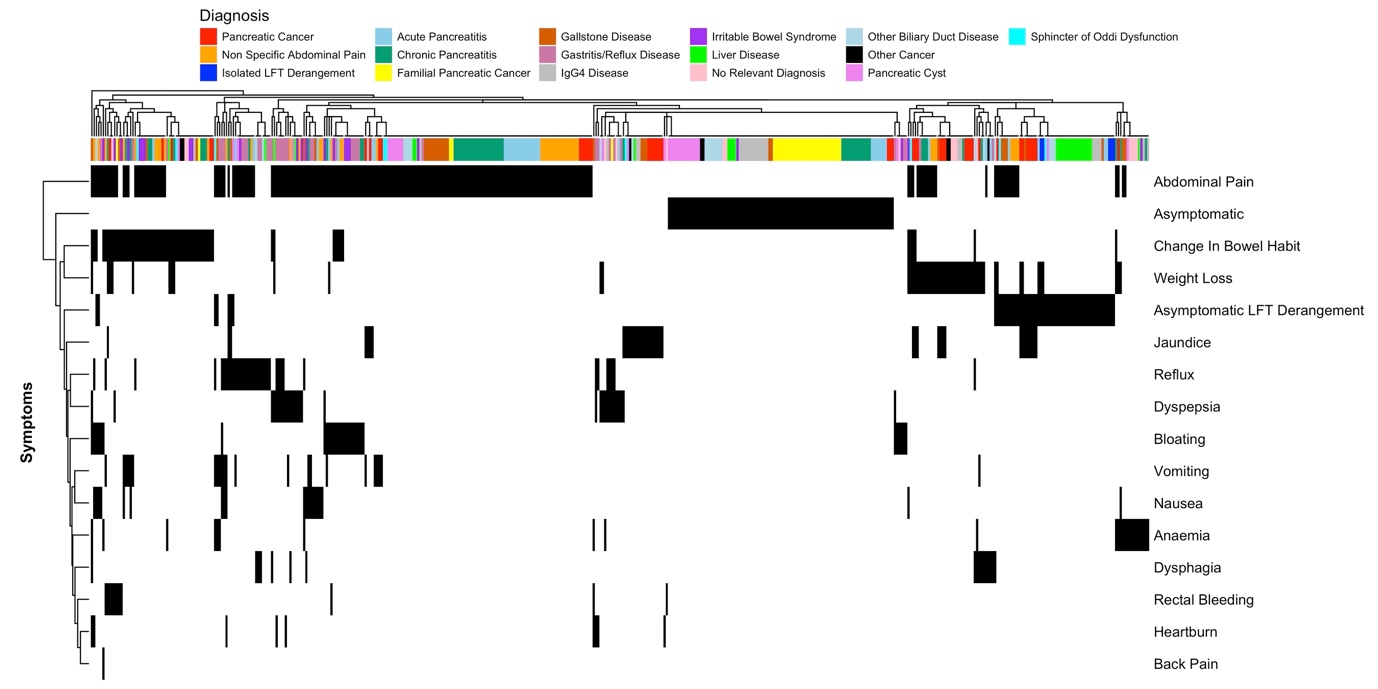
**

**Fig E. Performance of individual base-learner classifiers and stack ensemble for a reduced set of biomarkers, CA19-9, Age, Diabetes, VWF, CPE, CTSV, CEACAM1 and CD160. A** Base-learner performance in the discovery. Each base-learner classifier was developed by training with a recursive feature elimination technique (RFE) and logistic regression (glm) in samples belonging to each specific diagnosis class against the same 24 PDACs in the discovery set. The performance reported in A is, nevertheless, of each classifier in the whole discovery set. The performances reported in **B** correspond to the base-learners developed in the discovery set but applied to the whole validation set. In **C** and **D** the performance of an ensemble GLM stack based on the base-learners presented in A and B is reported in the discovery and held-out validation sets, respectively. The ROC AUC significance threshold is represented by a purple dashed line at 0.5. Error bars in figures correspond to 95% Confidence Intervals (CI), calculated by stratified bootstrapping 2000 times. See Methods for further details.

**
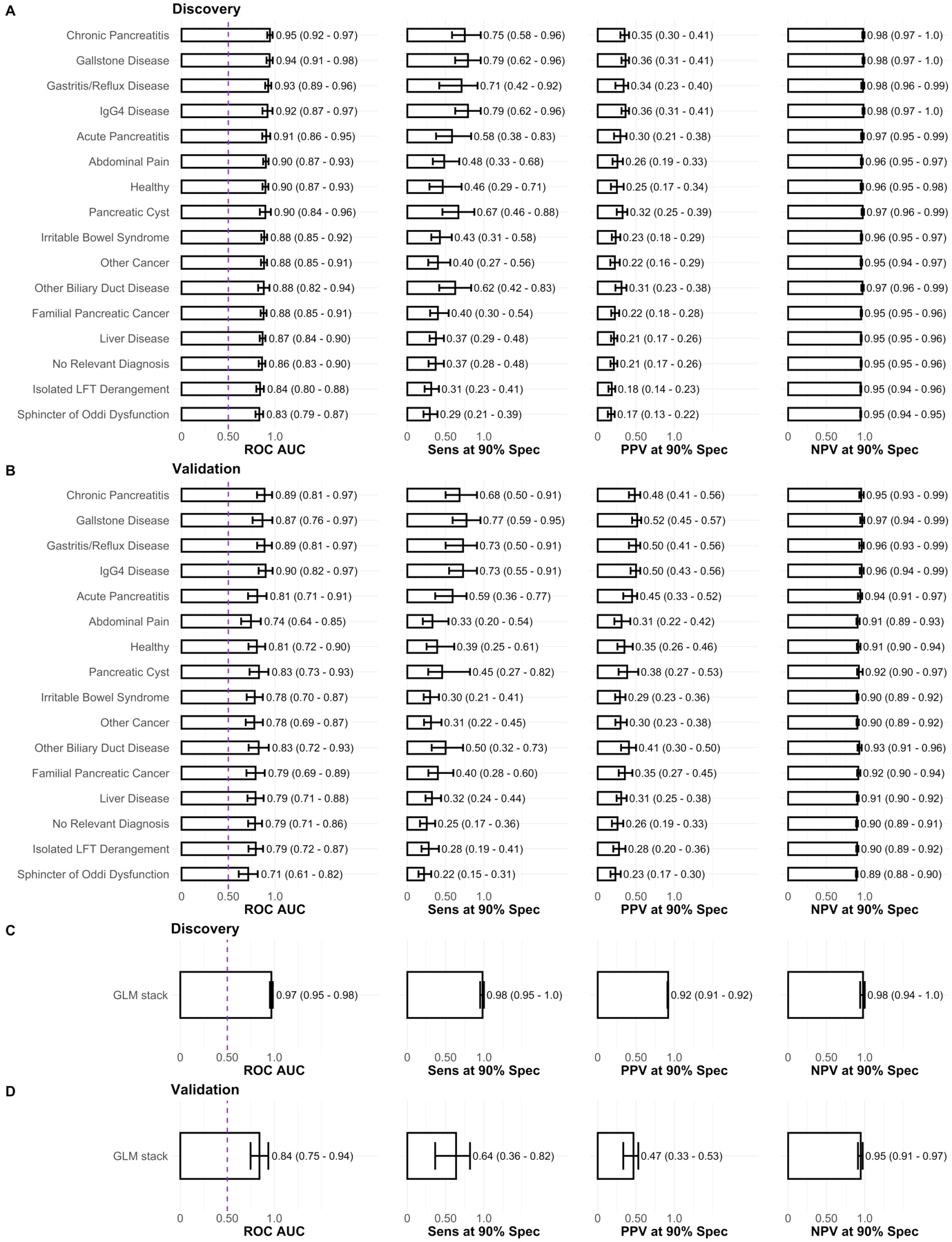
**

**Fig F. Feature importance for a reduced 8-marker model following the same principles as those described in Fig 2A and 2B.** The reduced set of biomarkers was CA19-9, Age, Diabetes, VWF, CPE, CTSV, CEACAM1 and CD160. See also performances in Fig E (in S1 Appendix). Selected features are ranked from left to right according to the average scaled importance across base learners. See Methods section in the main text for details.


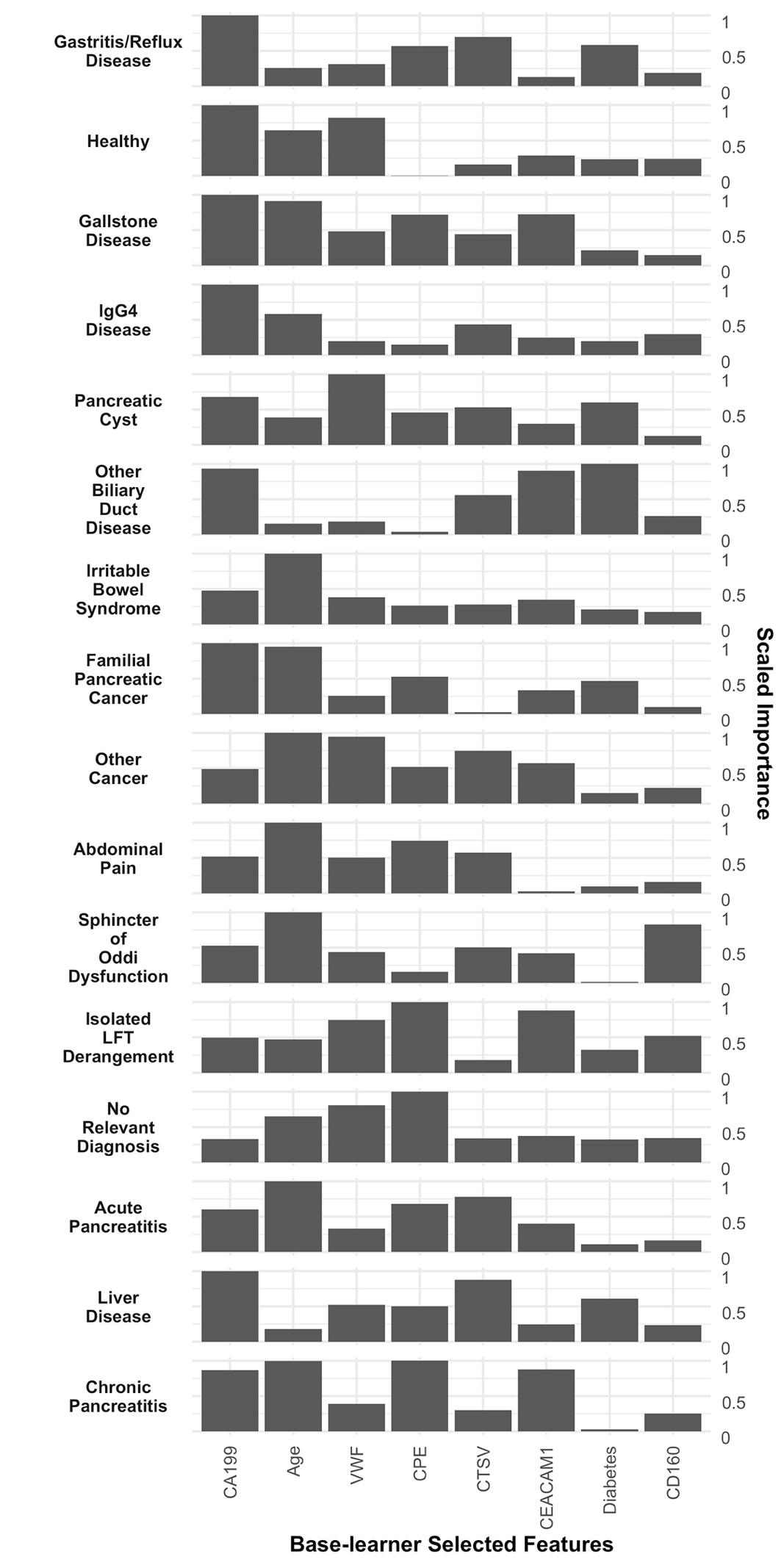


Supplementary Tables

Table A. Discovery set data and univariate association with PDAC status. Odds ratios (OR), 95% confidence intervals (CI) and p values were calculated according to a logistic regression model with a bias reduction method (see statistical section in Methods, main text).

| **Variable** | | **Cases** | | **Controls** | | **OR** | | **p value** | |
| --- | --- | --- | --- | --- | --- | --- | --- | --- | --- |
| Number of samples | | 24 | | 333 | | - | | - | |
| Mean age at sample draw (yr) (range) | | 70.79 (43.00-91.00) | | 58.39(19.00-89.00) | | 1.07 (1.03 - 1.11) | | 4.68e-05 | |
| Mean BMI (kg/m2) (range) | | 25.46 (19.81-41.35) | | 25.41 (15.22-42.19) | | 1.01 (0.89-1.12) | | 0.87 | |
| Gender | |  | |  | | 4.98 (2.08 – 13.50) | |  | |
| Male | | 18 | | 121 | |  |  | 0.00023 | |
| Female | | 6 | | 212 | |  |  |  |  |
| Diabetes |  |  |  |  |  |  |  |  |  |
| yes | | 5 | | 52 | | 1.51 (0.51 – 3.84) | | 0.43 | |
| no | | 281 | | 19 | |  |  |  |  |
| Ethnicity | |  | |  | |  | |  | |
| Caucasian | | 11 | | 189 | | 1.17 (0.70 – 2.02) | | 0.56 | |
| Unknown | | 9 | | 99 | |  |  |  |  |
| Asian | | 3 | | 15 | |  |  |  |  |
| Other | | 1 | | 13 | |  |  |  |  |
| Afro/Caribbean | | 0 | | 17 | |  |  |  |  |

Table B. Validation set data and univariate association with PDAC status. Odds ratios (OR), 95% confidence intervals (CI) and p values were calculated according to a logistic regression model with a bias reduction method (see statistical analysis section in Methods in the main text).

| **Variable** | | **Cases** | | **Controls** | | **OR** | | **p value** | |
| --- | --- | --- | --- | --- | --- | --- | --- | --- | --- |
| Number of Samples | | 23 | | 159 | | - | | - | |
| Mean age at sample draw (yr) (range) | | 68.61 (53.00 - 83.00) | | 57.94 (21.00 - 93.00) | | 1.06 (1.02 -1.04) | | 0.00071 | |
| Mean BMI (kg/m2) (range) | | 24.18 (12.04 - 31.62) | | 25.64 (17.60 - 38.30) | | 0.90 (0.78-1.01) | | 0.080 | |
| Gender | |  | |  | | 2.65 (1.11 – 6.58) | |  | |
| Male | | 14 | | 58 | |  |  | 0.028 | |
| Female | | 9 | | 101 | |  |  |  |  |
| Diabetes |  |  |  |  |  |  |  |  |  |
| yes | | 5 | | 52 | | 1.57 (0.51 – 4.23) | | 0.41 | |
| no | | 281 | | 19 | |  |  |  |  |
| Ethnicity | |  | |  | |  | |  | |
| Caucasian | | 10 | | 102 | | 2.66 (1.42 – 5.17) | | 0.0020 | |
| Unknown | | 12 | | 32 | |  |  |  |  |
| Asian | | 3 | | 15 | |  |  |  |  |
| Other | | 1 | | 5 | |  |  |  |  |
| Afro/Caribbean | | 0 | | 5 | |  |  |  |  |

Table C. Odds ratios, p values, ROC AUC, sensitivity (Sens), positive predictive value (PPV) and negative predictive value (NPV) for univariate logistic regression models derived in the discovery set. The performances of these model in the validation are presented in Table D (in S1 Appendix). Markers are ranked according to the p values. Spec: specificity.

| **Marker** | **OR** | **p value** | **ROC AUC** | **Sens at 90% Spec** | **PPV at 90% Spec** | **NPV at 90% Spec** |
| --- | --- | --- | --- | --- | --- | --- |
| CA19 - 9 | 1.85 (1.54 - 2.26) | 1.87E-12 | 0.79 (0.66 - 0.91) | 0.67 (0.5 - 0.83) | 0.32 (0.26 - 0.38) | 0.97 (0.96 - 0.99) |
| KITLG | 0.23 (0.14 - 0.37) | 7.95E-11 | 0.79 (0.68 - 0.89) | 0.5 (0.29 - 0.71) | 0.26 (0.17 - 0.34) | 0.96 (0.95 - 0.98) |
| CRP | 2.01 (1.59 - 2.61) | 4.12E-10 | 0.84 (0.74 - 0.92) | 0.58 (0.38 - 0.83) | 0.34 (0.25 - 0.42) | 0.96 (0.94 - 0.98) |
| VWF | 3.92 (2.28 - 7.78) | 1.89E-09 | 0.84 (0.76 - 0.9) | 0.5 (0.29 - 0.71) | 0.26 (0.17 - 0.34) | 0.96 (0.95 - 0.98) |
| SDC1 | 4.42 (2.56 - 8.01) | 8.47E-08 | 0.79 (0.66 - 0.89) | 0.67 (0.46 - 0.83) | 0.32 (0.25 - 0.38) | 0.97 (0.96 - 0.99) |
| KDR | 0.01 (0.00 - 0.07) | 2.02E-07 | 0.8 (0.72 - 0.87) | 0.42 (0.21 - 0.67) | 0.23 (0.13 - 0.32) | 0.96 (0.94 - 0.97) |
| TBIL | 2.03 (1.55 - 2.75) | 2.53E-07 | 0.72 (0.6 - 0.85) | 0.54 (0.29 - 0.71) | 0.32 (0.2 - 0.38) | 0.96 (0.94 - 0.97) |
| TNFRSF6B | 2.62 (1.80 - 3.94) | 3.12E-07 | 0.75 (0.64 - 0.86) | 0.5 (0.29 - 0.71) | 0.26 (0.17 - 0.34) | 0.96 (0.95 - 0.98) |
| FASLG | 0.20 (0.10 - 0.39) | 2.39E-06 | 0.77 (0.65 - 0.87) | 0.46 (0.21 - 0.67) | 0.25 (0.13 - 0.32) | 0.96 (0.94 - 0.97) |
| ESM-1 | 4.84 (2.35 - 10.69) | 5.99E-06 | 0.76 (0.66 - 0.84) | 0.29 (0.12 - 0.5) | 0.17 (0.08 - 0.27) | 0.95 (0.93 - 0.96) |
| EPHA2 | 5.15 (2.56 - 10.97) | 6.31E-06 | 0.73 (0.63 - 0.82) | 0.29 (0.12 - 0.46) | 0.17 (0.08 - 0.25) | 0.95 (0.93 - 0.96) |
| RET | 0.18 (0.08 - 0.38) | 7.01E-06 | 0.78 (0.68 - 0.87) | 0.42 (0.21 - 0.62) | 0.23 (0.13 - 0.31) | 0.96 (0.94 - 0.97) |
| TGFA | 0.33 (0.18 - 0.55) | 9.61E-06 | 0.79 (0.69 - 0.87) | 0.33 (0.17 - 0.62) | 0.19 (0.11 - 0.31) | 0.95 (0.94 - 0.97) |
| MUC16 | 2.39 (1.63 - 3.65) | 1.02E-05 | 0.72 (0.59 - 0.83) | 0.38 (0.21 - 0.58) | 0.21 (0.13 - 0.3) | 0.95 (0.94 - 0.97) |
| ITGAV | 0.05 (0.01 - 0.19) | 1.29E-05 | 0.78 (0.68 - 0.87) | 0.46 (0.17 - 0.67) | 0.25 (0.11 - 0.32) | 0.96 (0.94 - 0.97) |
| EGF | 0.55 (0.41 - 0.72) | 3.77E-05 | 0.79 (0.71 - 0.87) | 0.5 (0.21 - 0.71) | 0.26 (0.13 - 0.34) | 0.96 (0.94 - 0.98) |
| ICOSLG | 0.04 (0.01 - 0.20) | 5.60E-05 | 0.77 (0.66 - 0.85) | 0.29 (0.12 - 0.5) | 0.17 (0.08 - 0.26) | 0.95 (0.93 - 0.96) |
| CEACAM5 | 2.05 (1.46 - 2.94) | 6.15E-05 | 0.72 (0.61 - 0.83) | 0.33 (0.17 - 0.58) | 0.19 (0.11 - 0.3) | 0.95 (0.94 - 0.97) |
| CTSV | 0.27 (0.13 - 0.54) | 0.00019 | 0.73 (0.61 - 0.84) | 0.29 (0.08 - 0.5) | 0.17 (0.06 - 0.26) | 0.95 (0.93 - 0.96) |
| TFPI2 | 2.91 (1.65 - 5.38) | 0.00024 | 0.7 (0.58 - 0.8) | 0.29 (0.08 - 0.5) | 0.17 (0.06 - 0.26) | 0.95 (0.93 - 0.96) |
| WIF1 | 0.17 (0.06 - 0.45) | 0.00031 | 0.72 (0.61 - 0.82) | 0.33 (0.12 - 0.5) | 0.19 (0.08 - 0.26) | 0.95 (0.93 - 0.96) |
| TXLNA | 2.21 (1.41 - 3.51) | 0.00065 | 0.69 (0.55 - 0.81) | 0.42 (0.17 - 0.62) | 0.23 (0.11 - 0.31) | 0.96 (0.94 - 0.97) |
| LYPD3 | 0.24 (0.10 - 0.57) | 0.0012 | 0.68 (0.57 - 0.79) | 0.33 (0.17 - 0.58) | 0.19 (0.11 - 0.3) | 0.95 (0.94 - 0.97) |
| CA9 | 2.21 (1.33 - 3.68) | 0.0022 | 0.65 (0.52 - 0.77) | 0.29 (0.12 - 0.5) | 0.17 (0.08 - 0.26) | 0.95 (0.93 - 0.96) |
| FCRLB | 2.24 (1.32 - 3.74) | 0.0033 | 0.66 (0.53 - 0.78) | 0.38 (0.17 - 0.58) | 0.21 (0.11 - 0.3) | 0.95 (0.94 - 0.97) |
| TCL1A | 0.54 (0.35 - 0.82) | 0.0033 | 0.68 (0.57 - 0.78) | 0.29 (0.12 - 0.5) | 0.17 (0.08 - 0.26) | 0.95 (0.93 - 0.96) |
| MDK | 2.42 (1.34 - 4.42) | 0.0039 | 0.66 (0.54 - 0.77) | 0.25 (0.08 - 0.46) | 0.15 (0.06 - 0.25) | 0.94 (0.93 - 0.96) |
| CDKN1A | 1.82 (1.19 - 2.75) | 0.0066 | 0.63 (0.48 - 0.76) | 0.33 (0.12 - 0.5) | 0.19 (0.08 - 0.26) | 0.95 (0.93 - 0.96) |
| FURIN | 0.25 (0.09 - 0.70) | 0.0083 | 0.67 (0.54 - 0.79) | 0.29 (0.08 - 0.5) | 0.17 (0.06 - 0.26) | 0.95 (0.93 - 0.96) |
| TNFSF13 | 4.17 (1.43 - 12.15) | 0.0093 | 0.64 (0.54 - 0.75) | 0.25 (0.08 - 0.46) | 0.15 (0.06 - 0.25) | 0.94 (0.93 - 0.96) |
| GAL | 0.16 (0.04 - 0.65) | 0.011 | 0.63 (0.51 - 0.74) | 0.25 (0.08 - 0.46) | 0.15 (0.06 - 0.25) | 0.94 (0.93 - 0.96) |
| TNFSF10 | 0.24 (0.08 - 0.72) | 0.011 | 0.67 (0.55 - 0.77) | 0.17 (0.04 - 0.38) | 0.11 (0.03 - 0.21) | 0.94 (0.93 - 0.95) |
| CPE | 0.31 (0.12 - 0.77) | 0.012 | 0.66 (0.54 - 0.77) | 0.25 (0.08 - 0.46) | 0.15 (0.06 - 0.25) | 0.94 (0.93 - 0.96) |
| WFDC2 | 3.02 (1.27 - 7.34) | 0.012 | 0.64 (0.53 - 0.75) | 0.21 (0.04 - 0.42) | 0.13 (0.03 - 0.23) | 0.94 (0.93 - 0.96) |
| PPY | 0.74 (0.59 - 0.94) | 0.014 | 0.67 (0.56 - 0.78) | 0.21 (0.04 - 0.46) | 0.13 (0.03 - 0.25) | 0.94 (0.93 - 0.96) |
| METAP2 | 1.76 (1.12 - 2.77) | 0.015 | 0.6 (0.44 - 0.74) | 0.29 (0.12 - 0.5) | 0.17 (0.08 - 0.26) | 0.95 (0.93 - 0.96) |
| XPNPEP2 | 0.70 (0.50 - 0.93) | 0.018 | 0.6 (0.49 - 0.7) | 0.17 (0.04 - 0.33) | 0.11 (0.03 - 0.19) | 0.94 (0.93 - 0.95) |
| NECTIN4 | 2.71 (1.19 - 6.04) | 0.018 | 0.61 (0.5 - 0.72) | 0.21 (0.04 - 0.38) | 0.13 (0.03 - 0.21) | 0.94 (0.93 - 0.95) |
| Creatinine | 0.29 (0.10 - 0.86) | 0.024 | 0.62 (0.49 - 0.73) | 0.25 (0.08 - 0.42) | 0.18 (0.07 - 0.27) | 0.93 (0.92 - 0.95) |
| KLK8 | 0.34 (0.13 - 0.88) | 0.026 | 0.61 (0.47 - 0.74) | 0.35 (0.08 - 0.54) | 0.2 (0.06 - 0.28) | 0.95 (0.93 - 0.96) |
| CD207 | 0.35 (0.13 - 0.89) | 0.028 | 0.62 (0.51 - 0.72) | 0.12 (0 - 0.29) | 0.08 (0 - 0.17) | 0.93 (0.93 - 0.95) |
| MIA | 0.24 (0.07 - 0.92) | 0.038 | 0.62 (0.49 - 0.73) | 0.25 (0.08 - 0.42) | 0.15 (0.06 - 0.23) | 0.94 (0.93 - 0.96) |
| GPNMB | 9.28 (1.10 - 84.13) | 0.040 | 0.63 (0.52 - 0.74) | 0.17 (0.04 - 0.38) | 0.11 (0.03 - 0.21) | 0.94 (0.93 - 0.95) |
| ERBB2 | 3.21 (1.06 - 9.50) | 0.040 | 0.56 (0.41 - 0.71) | 0.33 (0.17 - 0.54) | 0.19 (0.11 - 0.28) | 0.95 (0.94 - 0.96) |
| DLL1 | 3.06 (1.05 - 9.11) | 0.041 | 0.58 (0.45 - 0.7) | 0.25 (0.08 - 0.46) | 0.15 (0.06 - 0.25) | 0.94 (0.93 - 0.96) |
| TNFRSF19 | 0.45 (0.20 - 0.98) | 0.044 | 0.65 (0.51 - 0.77) | 0.33 (0.08 - 0.54) | 0.19 (0.06 - 0.28) | 0.95 (0.93 - 0.96) |
| AR | 1.47 (1.00 - 2.09) | 0.053 | 0.63 (0.51 - 0.74) | 0.21 (0.04 - 0.38) | 0.13 (0.03 - 0.21) | 0.94 (0.93 - 0.95) |
| NT5E | 1.76 (0.99 - 3.08) | 0.053 | 0.63 (0.51 - 0.74) | 0.17 (0.04 - 0.46) | 0.11 (0.03 - 0.25) | 0.94 (0.93 - 0.96) |
| THBS2 | 1.60 (0.98 - 2.69) | 0.060 | 0.62 (0.48 - 0.75) | 0.25 (0.08 - 0.46) | 0.15 (0.06 - 0.25) | 0.94 (0.93 - 0.96) |
| ADAM8 | 0.42 (0.16 - 1.04) | 0.060 | 0.6 (0.48 - 0.73) | 0.25 (0.08 - 0.46) | 0.15 (0.06 - 0.25) | 0.94 (0.93 - 0.96) |
| GZMB | 0.57 (0.30 - 1.06) | 0.077 | 0.61 (0.48 - 0.74) | 0.25 (0.08 - 0.5) | 0.15 (0.06 - 0.26) | 0.94 (0.93 - 0.96) |
| IGF1R | 0.37 (0.12 - 1.12) | 0.078 | 0.64 (0.52 - 0.77) | 0.29 (0.08 - 0.5) | 0.17 (0.06 - 0.26) | 0.95 (0.93 - 0.96) |
| PKM | 1.12 (0.98 - 1.30) | 0.10 | 0.57 (0.43 - 0.72) | 0.12 (0 - 0.46) | 0.1 (0 - 0.29) | 0.92 (0.91 - 0.95) |
| KLK14 | 0.53 (0.25 - 1.16) | 0.11 | 0.59 (0.48 - 0.7) | 0.12 (0 - 0.29) | 0.08 (0 - 0.17) | 0.93 (0.93 - 0.95) |
| ERBB3 | 0.32 (0.08 - 1.32) | 0.11 | 0.59 (0.46 - 0.72) | 0.25 (0.08 - 0.42) | 0.15 (0.06 - 0.23) | 0.94 (0.93 - 0.96) |
| CRNN | 0.67 (0.40 - 1.12) | 0.13 | 0.64 (0.53 - 0.75) | 0.21 (0.04 - 0.42) | 0.13 (0.03 - 0.23) | 0.94 (0.93 - 0.96) |
| FOLR3 | 0.85 (0.64 - 1.05) | 0.14 | 0.57 (0.47 - 0.68) | 0.12 (0 - 0.29) | 0.08 (0 - 0.17) | 0.93 (0.93 - 0.95) |
| CXCL13 | 0.59 (0.28 - 1.18) | 0.14 | 0.58 (0.43 - 0.71) | 0.29 (0.12 - 0.5) | 0.17 (0.08 - 0.26) | 0.95 (0.93 - 0.962) |
| SEZ6L | 0.39 (0.12 - 1.39) | 0.15 | 0.63 (0.5 - 0.75) | 0.29 (0.04 - 0.46) | 0.17 (0.03 - 0.25) | 0.95 (0.93 - 0.96) |
| KLK13 | 0.67 (0.38 - 1.17) | 0.16 | 0.58 (0.45 - 0.72) | 0.25 (0.08 - 0.46) | 0.15 (0.06 - 0.25) | 0.94 (0.93 - 0.96) |
| S100A11 | 0.57 (0.23 - 1.24) | 0.16 | 0.54 (0.42 - 0.66) | 0.21 (0.08 - 0.38) | 0.13 (0.06 - 0.21) | 0.94 (0.93 - 0.95) |
| ITGB5 | 2.09 (0.72 - 6.18) | 0.18 | 0.46 (0.33 - 0.59) | 0.12 (0 - 0.25) | 0.08 (0 - 0.15) | 0.93 (0.93 - 0.94) |
| IL6 | 1.18 (0.91 - 1.46) | 0.18 | 0.61 (0.5 - 0.72) | 0.21 (0.04 - 0.38) | 0.13 (0.03 - 0.21) | 0.94 (0.93 - 0.95) |
| MICA/B | 1.24 (0.91 - 1.88) | 0.19 | 0.62 (0.49 - 0.74) | 0.25 (0.08 - 0.46) | 0.15 (0.06 - 0.25) | 0.94 (0.93 - 0.96) |
| CXCL17 | 1.40 (0.84 - 2.30) | 0.19 | 0.55 (0.46 - 0.65) | 0.08 (0 - 0.21) | 0.06 (0 - 0.13) | 0.93 (0.93 - 0.94) |
| CD70 | 0.62 (0.30 - 1.27) | 0.20 | 0.58 (0.46 - 0.69) | 0.12 (0 - 0.29) | 0.08 (0 - 0.17) | 0.93 (0.93 - 0.95) |
| CD160 | 0.63 (0.30 - 1.28) | 0.20 | 0.57 (0.43 - 0.7) | 0.25 (0.08 - 0.46) | 0.15 (0.06 - 0.25) | 0.94 (0.93 - 0.96) |
| TLR3 | 0.70 (0.41 - 1.22) | 0.20 | 0.59 (0.44 - 0.72) | 0.17 (0 - 0.38) | 0.11 (0 - 0.21) | 0.94 (0.93 - 0.95) |
| VIM | 0.81 (0.55 - 1.17) | 0.27 | 0.55 (0.44 - 0.66) | 0.12 (0 - 0.29) | 0.08 (0 - 0.17) | 0.93 (0.93 - 0.95) |
| IFNGR1 | 1.99 (0.53 - 6.82) | 0.30 | 0.53 (0.39 - 0.65) | 0.12 (0 - 0.29) | 0.08 (0 - 0.17) | 0.93 (0.93 - 0.95) |
| FGFBP1 | 1.70 (0.59 - 4.53) | 0.32 | 0.54 (0.39 - 0.68) | 0.21 (0.08 - 0.46) | 0.13 (0.06 - 0.25) | 0.94 (0.93 - 0.96) |
| LY9 | 0.64 (0.24 - 1.70) | 0.36 | 0.56 (0.43 - 0.69) | 0.25 (0.08 - 0.46) | 0.15 (0.06 - 0.25) | 0.94 (0.93 - 0.96) |
| FOLR1 | 0.68 (0.28 - 1.65) | 0.39 | 0.59 (0.46 - 0.7) | 0.04 (0 - 0.33) | 0.03 (0 - 0.19) | 0.93 (0.93 - 0.95) |
| MSLN | 1.25 (0.74 - 2.05) | 0.40 | 0.58 (0.49 - 0.66) | 0.04 (0 - 0.17) | 0.03 (0 - 0.11) | 0.93 (0.93 - 0.94) |
| PODXL | 2.39 (0.31 - 18.29) | 0.40 | 0.53 (0.4 - 0.67) | 0.21 (0.04 - 0.42) | 0.13 (0.03 - 0.23) | 0.94 (0.93 - 0.96) |
| GZMH | 1.23 (0.74 - 1.95) | 0.41 | 0.47 (0.34 - 0.6) | 0.04 (0 - 0.17) | 0.03 (0 - 0.11) | 0.93 (0.93 - 0.94) |
| S100A4 | 0.81 (0.46 - 1.34) | 0.42 | 0.57 (0.42 - 0.7) | 0.33 (0.17 - 0.5) | 0.19 (0.11 - 0.27) | 0.95 (0.94 - 0.96) |
| ERBB4 | 0.64 (0.20 - 2.08) | 0.45 | 0.57 (0.43 - 0.69) | 0.12 (0 - 0.38) | 0.08 (0 - 0.21) | 0.93 (0.93 - 0.95) |
| TGFBR2 | 0.71 (0.28 - 1.75) | 0.46 | 0.59 (0.45 - 0.71) | 0.12 (0 - 0.29) | 0.08 (0 - 0.17) | 0.93 (0.93 - 0.95) |
| SMAD5 | 0.57 (0.15 - 2.95) | 0.46 | 0.61 (0.52 - 0.71) | 0.04 (0 - 0.12) | 0.03 (0 - 0.08) | 0.93 (0.93 - 0.94) |
| VEGFA | 0.78 (0.38 - 1.57) | 0.49 | 0.52 (0.39 - 0.65) | 0.17 (0.04 - 0.33) | 0.11 (0.03 - 0.19) | 0.94 (0.93 - 0.95) |
| CD48 | 0.66 (0.19 - 2.29) | 0.50 | 0.53 (0.4 - 0.66) | 0.21 (0.04 - 0.38) | 0.13 (0.03 - 0.21) | 0.94 (0.93 - 0.95) |
| RSPO3 | 1.16 (0.67 - 1.86) | 0.57 | 0.49 (0.38 - 0.59) | 0 (0 - 0) | 0 (0 - 0) | 0.93 (0.93 - 0.93) |
| CCN4 | 0.82 (0.38 - 1.64) | 0.59 | 0.55 (0.42 - 0.68) | 0.17 (0.04 - 0.33) | 0.11 (0.03 - 0.19) | 0.94 (0.93 - 0.95) |
| IL6ST | 0.87 (0.60 - 2.13) | 0.59 | 0.66 (0.52 - 0.8) | 0.38 (0.21 - 0.58) | 0.21 (0.13 - 0.3) | 0.95 (0.94 - 0.97) |
| LYN | 1.22 (0.39 - 2.70) | 0.69 | 0.53 (0.38 - 0.67) | 0.25 (0.08 - 0.42) | 0.15 (0.06 - 0.23) | 0.94 (0.93 - 0.96) |
| SCAMP3 | 0.94 (0.68 - 1.26) | 0.70 | 0.5 (0.38 - 0.62) | 0 (0 - 0) | 0 (0 - 0) | 0.93 (0.93 - 0.93) |
| CCN1 | 1.11 (0.62 - 2.02) | 0.72 | 0.52 (0.4 - 0.63) | 0 (0 - 0) | 0 (0 - 0) | 0.93 (0.93 - 0.93) |
| SPARC | 0.61 (0.05 - 9.54) | 0.72 | 0.48 (0.37 - 0.59) | 0 (0 - 0) | 0 (0 - 0) | 0.93 (0.93 - 0.93) |
| TNFRSF4 | 1.13 (0.55 - 2.19) | 0.73 | 0.5 (0.37 - 0.64) | 0.17 (0.04 - 0.38) | 0.11 (0.03 - 0.21) | 0.94 (0.93 - 0.95) |
| ABL1 | 0.92 (0.55 - 1.46) | 0.75 | 0.48 (0.34 - 0.6) | 0.08 (0 - 0.21) | 0.06 (0 - 0.13) | 0.93 (0.93 - 0.94) |
| CEACAM1 | 0.83 (0.34 - 4.06) | 0.75 | 0.54 (0.42 - 0.65) | 0.17 (0 - 0.29) | 0.11 (0 - 0.17) | 0.94 (0.93 - 0.95) |
| FADD | 1.07 (0.65 - 1.64) | 0.79 | 0.55 (0.42 - 0.66) | 0.08 (0 - 0.25) | 0.06 (0 - 0.15) | 0.93 (0.93 - 0.94) |
| ADAMTS15 | 1.09 (0.58 - 2.06) | 0.79 | 0.51 (0.39 - 0.64) | 0.12 (0 - 0.25) | 0.08 (0 - 0.15) | 0.93 (0.93 - 0.943) |
| CD27 | 1.09 (0.45 - 2.58) | 0.84 | 0.52 (0.39 - 0.65) | 0.12 (0 - 0.29) | 0.08 (0 - 0.17) | 0.93 (0.93 - 0.95) |
| HGF | 1.05 (0.47 - 2.22) | 0.91 | 0.55 (0.41 - 0.7) | 0.25 (0.08 - 0.42) | 0.15 (0.06 - 0.23) | 0.94 (0.93 - 0.96) |
| KLK11 | 0.96 (0.40 - 2.23) | 0.92 | 0.49 (0.39 - 0.61) | 0.08 (0 - 0.25) | 0.06 (0 - 0.15) | 0.93 (0.93 - 0.94) |
| ANXA1 | 0.98 (0.63 - 1.51) | 0.94 | 0.51 (0.39 - 0.63) | 0.04 (0 - 0.17) | 0.03 (0 - 0.11) | 0.93 (0.93 - 0.94) |
| FLT4 | 0.95 (0.18 - 5.70) | 0.96 | 0.52 (0.4 - 0.65) | 0.08 (0 - 0.21) | 0.06 (0 - 0.13) | 0.93 (0.93 - 0.94) |
| GPC1 | 1.00 (0.37 - 2.75) | 1.00 | 0.54 (0.41 - 0.67) | 0.17 (0.04 - 0.33) | 0.11 (0.03 - 0.19) | 0.94 (0.93 - 0.95) |

Table D. ROC AUC, sensitivity (Sens), positive predictive value (PPV) and negative predictive value (NPV) in the validation set of the univariate logistic regression model derived in the discovery set. Markers are ranked according to their respective p values in the discovery set (see Table C in S1 Appendix). Spec: specificity.

| **Marker** | **ROC AUC** | **Sens at 90% Spec** | **PPV at 90% Spec** | **NPV at 90% Spec** |
| --- | --- | --- | --- | --- |
| CA19 - 9 | 0.80 (0.66 - 0.93) | 0.65 (0.48 - 0.87) | 0.49 (0.41 - 0.56) | 0.95 (0.92 - 0.98) |
| KITLG | 0.82 (0.72 - 0.90) | 0.48 (0.22 - 0.70) | 0.41 (0.24 - 0.50) | 0.92 (0.89 - 0.95) |
| CRP | 0.85 (0.76 - 0.92) | 0.59 (0.32 - 0.86) | 0.47 (0.32 - 0.56) | 0.94 (0.90 - 0.98) |
| VWF | 0.74 (0.64 - 0.84) | 0.26 (0.04 - 0.52) | 0.27 (0.06 - 0.43) | 0.89 (0.87 - 0.93) |
| SDC1 | 0.72 (0.59 - 0.83) | 0.30 (0.09 - 0.61) | 0.31 (0.11 - 0.47) | 0.90 (0.87 - 0.94) |
| KDR | 0.77 (0.67 - 0.86) | 0.39 (0.17 - 0.61) | 0.36 (0.20 - 0.47) | 0.91 (0.88 - 0.94) |
| TBIL | 0.67 (0.55 - 0.77) | 0.23 (0.09 - 0.41) | 0.25 (0.12 - 0.38) | 0.89 (0.87 - 0.91) |
| TNFRSF6B | 0.72 (0.60 - 0.83) | 0.39 (0.13 - 0.61) | 0.36 (0.16 - 0.47) | 0.91 (0.88 - 0.94) |
| FASLG | 0.74 (0.64 - 0.84) | 0.26 (0.09 - 0.52) | 0.27 (0.11 - 0.43) | 0.89 (0.87 - 0.93) |
| ESM1 | 0.73 (0.62 - 0.82) | 0.30 (0.13 - 0.52) | 0.31 (0.16 - 0.43) | 0.90 (0.88 - 0.93) |
| EPHA2 | 0.70 (0.56 - 0.82) | 0.39 (0.17 - 0.61) | 0.36 (0.20 - 0.47) | 0.91 (0.88 - 0.94) |
| RET | 0.69 (0.59 - 0.78) | 0.26 (0.09 - 0.43) | 0.27 (0.11 - 0.39) | 0.89 (0.87 - 0.92) |
| TGFA | 0.71 (0.59 - 0.81) | 0.26 (0.09 - 0.48) | 0.27 (0.11 - 0.41) | 0.89 (0.87 - 0.92) |
| MUC16 | 0.71 (0.58 - 0.84) | 0.48 (0.22 - 0.70) | 0.41 (0.24 - 0.50) | 0.92 (0.89 - 0.95) |
| ITGAV | 0.77 (0.67 - 0.85) | 0.39 (0.13 - 0.57) | 0.36 (0.16 - 0.45) | 0.91 (0.88 - 0.94) |
| EGF | 0.74 (0.61 - 0.85) | 0.52 (0.13 - 0.70) | 0.43 (0.16 - 0.50) | 0.93 (0.88 - 0.95) |
| ICOSLG | 0.69 (0.59 - 0.79) | 0.30 (0.09 - 0.48) | 0.31 (0.11 - 0.41) | 0.90 (0.87 - 0.92) |
| CEACAM5 | 0.72 (0.60 - 0.83) | 0.35 (0.13 - 0.52) | 0.33 (0.16 - 0.43) | 0.91 (0.88 - 0.93) |
| CTSV | 0.74 (0.63 - 0.84) | 0.26 (0.09 - 0.57) | 0.27 (0.11 - 0.45) | 0.89 (0.87 - 0.94) |
| TFPI2 | 0.67 (0.54 - 0.78) | 0.22 (0.04 - 0.48) | 0.24 (0.06 - 0.41) | 0.89 (0.87 - 0.92) |
| WIF1 | 0.63 (0.51 - 0.74) | 0.17 (0.00 - 0.39) | 0.20 (0.00 - 0.36) | 0.88 (0.86 - 0.91) |
| TXLNA | 0.64 (0.51 - 0.75) | 0.22 (0.09 - 0.43) | 0.24 (0.11 - 0.39) | 0.89 (0.87 - 0.92) |
| LYPD3 | 0.67 (0.54 - 0.79) | 0.22 (0.04 - 0.43) | 0.24 (0.06 - 0.39) | 0.89 (0.87 - 0.92) |
| CA9 | 0.73 (0.64 - 0.82) | 0.17 (0.04 - 0.35) | 0.20 (0.06 - 0.33) | 0.88 (0.87 - 0.91) |
| FCRLB | 0.69 (0.59 - 0.79) | 0.26 (0.04 - 0.43) | 0.27 (0.06 - 0.39) | 0.89 (0.87 - 0.92) |
| TCL1A | 0.58 (0.45 - 0.70) | 0.17 (0.04 - 0.39) | 0.20 (0.06 - 0.36) | 0.88 (0.87 - 0.91) |
| MDK | 0.70 (0.59 - 0.81) | 0.35 (0.17 - 0.57) | 0.33 (0.20 - 0.45) | 0.91 (0.88 - 0.94) |
| CDKN1A | 0.62 (0.49 - 0.74) | 0.13 (0.00 - 0.43) | 0.16 (0.00 - 0.39) | 0.88 (0.86 - 0.92) |
| FURIN | 0.64 (0.51 - 0.77) | 0.22 (0.04 - 0.48) | 0.24 (0.06 - 0.41) | 0.89 (0.87 - 0.92) |
| TNFSF13 | 0.64 (0.52 - 0.76) | 0.22 (0.04 - 0.43) | 0.24 (0.06 - 0.39) | 0.89 (0.87 - 0.92) |
| GAL | 0.61 (0.47 - 0.72) | 0.17 (0.04 - 0.39) | 0.20 (0.06 - 0.36) | 0.88 (0.87 - 0.91) |
| TNFSF10 | 0.66 (0.54 - 0.77) | 0.22 (0.04 - 0.39) | 0.24 (0.06 - 0.36) | 0.89 (0.87 - 0.91) |
| CPE | 0.64 (0.50 - 0.76) | 0.22 (0.04 - 0.43) | 0.24 (0.06 - 0.39) | 0.89 (0.87 - 0.92) |
| WFDC2 | 0.75 (0.62 - 0.86) | 0.30 (0.09 - 0.61) | 0.31 (0.11 - 0.47) | 0.90 (0.87 - 0.94) |
| PPY | 0.62 (0.51 - 0.73) | 0.17 (0.04 - 0.35) | 0.20 (0.06 - 0.33) | 0.88 (0.87 - 0.91) |
| METAP2 | 0.62 (0.47 - 0.75) | 0.26 (0.09 - 0.48) | 0.27 (0.11 - 0.41) | 0.89 (0.87 - 0.92) |
| XPNPEP2 | 0.57 (0.45 - 0.69) | 0.09 (0.00 - 0.30) | 0.11 (0.00 - 0.31) | 0.87 (0.86 - 0.90) |
| NECTIN4 | 0.62 (0.49 - 0.75) | 0.26 (0.04 - 0.48) | 0.27 (0.06 - 0.41) | 0.89 (0.87 - 0.92) |
| Creatinine | 0.44 (0.29 - 0.58) | 0.09 (0.00 - 0.27) | 0.12 (0.00 - 0.29) | 0.87 (0.86 - 0.89) |
| KLK8 | 0.65 (0.52 - 0.78) | 0.26 (0.04 - 0.48) | 0.27 (0.06 - 0.41) | 0.89 (0.87 - 0.92) |
| CD207 | 0.51 (0.38 - 0.64) | 0.04 (0.00 - 0.17) | 0.06 (0.00 - 0.20) | 0.87 (0.86 - 0.88) |
| MIA | 0.51 (0.40 - 0.63) | 0.04 (0.00 - 0.17) | 0.06 (0.00 - 0.20) | 0.87 (0.86 - 0.88) |
| GPNMB | 0.70 (0.58 - 0.81) | 0.30 (0.09 - 0.52) | 0.31 (0.11 - 0.43) | 0.90 (0.87 - 0.93) |
| ERBB2 | 0.47 (0.34 - 0.61) | 0.13 (0.00 - 0.30) | 0.16 (0.00 - 0.31) | 0.88 (0.86 - 0.90) |
| DLL1 | 0.65 (0.52 - 0.78) | 0.26 (0.04 - 0.52) | 0.27 (0.06 - 0.43) | 0.89 (0.87 - 0.93) |
| TNFRSF19 | 0.52 (0.39 - 0.65) | 0.13 (0.00 - 0.30) | 0.16 (0.00 - 0.31) | 0.88 (0.86 - 0.90) |
| AR | 0.70 (0.57 - 0.81) | 0.22 (0.04 - 0.52) | 0.24 (0.06 - 0.43) | 0.89 (0.87 - 0.93) |
| NT5E | 0.68 (0.55 - 0.80) | 0.22 (0.04 - 0.48) | 0.24 (0.06 - 0.41) | 0.89 (0.87 - 0.92) |
| THBS2 | 0.73 (0.61 - 0.84) | 0.43 (0.22 - 0.65) | 0.39 (0.24 - 0.49) | 0.92 (0.89 - 0.95) |
| ADAM8 | 0.50 (0.38 - 0.62) | 0.09 (0.00 - 0.26) | 0.11 (0.00 - 0.27) | 0.87 (0.86 - 0.89) |
| GZMB | 0.60 (0.47 - 0.73) | 0.22 (0.09 - 0.39) | 0.24 (0.11 - 0.36) | 0.89 (0.87 - 0.91) |
| IGF1R | 0.51 (0.38 - 0.64) | 0.09 (0.00 - 0.22) | 0.11 (0.00 - 0.24) | 0.87 (0.86 - 0.89) |
| PKM | 0.61 (0.46 - 0.75) | 0.23 (0.05 - 0.45) | 0.25 (0.06 - 0.40) | 0.89 (0.86 - 0.92) |
| KLK14 | 0.51 (0.37 - 0.65) | 0.22 (0.04 - 0.39) | 0.24 (0.06 - 0.36) | 0.89 (0.87 - 0.91) |
| ERBB3 | 0.61 (0.50 - 0.72) | 0.13 (0.00 - 0.35) | 0.16 (0.00 - 0.33) | 0.88 (0.86 - 0.91) |
| CRNN | 0.68 (0.57 - 0.78) | 0.17 (0.04 - 0.35) | 0.20 (0.06 - 0.33) | 0.88 (0.87 - 0.91) |
| FOLR3 | 0.54 (0.44 - 0.65) | 0.09 (0.00 - 0.22) | 0.11 (0.00 - 0.24) | 0.87 (0.86 - 0.89) |
| CXCL13 | 0.44 (0.31 - 0.58) | 0.00 (0.00 - 0.13) | 0.00 (0.00 - 0.16) | 0.86 (0.86 - 0.88) |
| SEZ6L | 0.61 (0.48 - 0.73) | 0.17 (0.04 - 0.35) | 0.20 (0.06 - 0.33) | 0.88 (0.87 - 0.91) |
| KLK13 | 0.63 (0.51 - 0.74) | 0.09 (0.00 - 0.26) | 0.11 (0.00 - 0.27) | 0.87 (0.86 - 0.89) |
| S100A11 | 0.58 (0.45 - 0.70) | 0.17 (0.00 - 0.35) | 0.20 (0.00 - 0.33) | 0.88 (0.86 - 0.91) |
| ITGB5 | 0.50 (0.36 - 0.62) | 0.00 (0.00 - 0.13) | 0.00 (0.00 - 0.16) | 0.86 (0.86 - 0.88) |
| IL6 | 0.69 (0.58 - 0.80) | 0.13 (0.00 - 0.39) | 0.16 (0.00 - 0.36) | 0.88 (0.86 - 0.91) |
| MICA/B | 0.58 (0.45 - 0.69) | 0.13 (0.00 - 0.30) | 0.16 (0.00 - 0.31) | 0.88 (0.86 - 0.90) |
| CXCL17 | 0.52 (0.41 - 0.64) | 0.04 (0.00 - 0.17) | 0.06 (0.00 - 0.20) | 0.87 (0.86 - 0.88) |
| CD70 | 0.49 (0.35 - 0.61) | 0.09 (0.00 - 0.26) | 0.11 (0.00 - 0.27) | 0.87 (0.86 - 0.89) |
| CD160 | 0.51 (0.36 - 0.65) | 0.09 (0.00 - 0.26) | 0.11 (0.00 - 0.27) | 0.87 (0.86 - 0.89) |
| TLR3 | 0.68 (0.54 - 0.80) | 0.43 (0.17 - 0.65) | 0.39 (0.20 - 0.49) | 0.92 (0.88 - 0.95) |
| VIM | 0.49 (0.36 - 0.62) | 0.13 (0.00 - 0.26) | 0.16 (0.00 - 0.27) | 0.88 (0.86 - 0.89) |
| IFNGR1 | 0.54 (0.40 - 0.69) | 0.13 (0.00 - 0.35) | 0.16 (0.00 - 0.33) | 0.88 (0.86 - 0.91) |
| FGFBP1 | 0.62 (0.49 - 0.75) | 0.22 (0.09 - 0.43) | 0.24 (0.11 - 0.39) | 0.89 (0.87 - 0.92) |
| LY9 | 0.49 (0.36 - 0.62) | 0.04 (0.00 - 0.17) | 0.06 (0.00 - 0.20) | 0.87 (0.86 - 0.88) |
| FOLR1 | 0.51 (0.37 - 0.63) | 0.09 (0.00 - 0.22) | 0.11 (0.00 - 0.24) | 0.87 (0.86 - 0.89) |
| MSLN | 0.67 (0.56 - 0.79) | 0.35 (0.00 - 0.52) | 0.33 (0.00 - 0.43) | 0.91 (0.86 - 0.93) |
| PODXL | 0.47 (0.34 - 0.61) | 0.09 (0.00 - 0.26) | 0.11 (0.00 - 0.27) | 0.87 (0.86 - 0.89) |
| GZMH | 0.47 (0.33 - 0.60) | 0.13 (0.00 - 0.30) | 0.16 (0.00 - 0.31) | 0.88 (0.86 - 0.90) |
| S100A4 | 0.54 (0.41 - 0.66) | 0.13 (0.00 - 0.26) | 0.16 (0.00 - 0.27) | 0.88 (0.86 - 0.89) |
| ERBB4 | 0.48 (0.34 - 0.60) | 0.09 (0.00 - 0.22) | 0.11 (0.00 - 0.24) | 0.87 (0.86 - 0.89) |
| TGFBR2 | 0.49 (0.36 - 0.63) | 0.00 (0.00 - 0.17) | 0.00 (0.00 - 0.20) | 0.86 (0.86 - 0.88) |
| SMAD5 | 0.62 (0.51 - 0.73) | 0.04 (0.00 - 0.22) | 0.06 (0.00 - 0.24) | 0.87 (0.86 - 0.89) |
| VEGFA | 0.53 (0.40 - 0.65) | 0.09 (0.00 - 0.26) | 0.11 (0.00 - 0.27) | 0.87 (0.86 - 0.89) |
| CD48 | 0.51 (0.39 - 0.64) | 0.04 (0.00 - 0.22) | 0.06 (0.00 - 0.24) | 0.87 (0.86 - 0.89) |
| RSPO3 | 0.48 (0.35 - 0.61) | 0.09 (0.00 - 0.26) | 0.11 (0.00 - 0.27) | 0.87 (0.86 - 0.89) |
| CCN4 | 0.62 (0.49 - 0.74) | 0.22 (0.04 - 0.43) | 0.24 (0.06 - 0.39) | 0.89 (0.87 - 0.92) |
| IL6ST | 0.68 (0.52 - 0.81) | 0.39 (0.22 - 0.61) | 0.36 (0.24 - 0.47) | 0.91 (0.89 - 0.94) |
| LYN | 0.52 (0.39 - 0.65) | 0.22 (0.04 - 0.39) | 0.24 (0.06 - 0.36) | 0.89 (0.87 - 0.91) |
| SCAMP3 | 0.49 (0.38 - 0.61) | 0.00 (0.00 - 0.17) | 0.00 (0.00 - 0.20) | 0.86 (0.86 - 0.88) |
| CCN1 | 0.50 (0.40 - 0.60) | 0.00 (0.00 - 0.04) | 0.00 (0.00 - 0.06) | 0.86 (0.86 - 0.87) |
| SPARC | 0.52 (0.40 - 0.64) | 0.04 (0.00 - 0.26) | 0.06 (0.00 - 0.27) | 0.87 (0.86 - 0.89) |
| TNFRSF4 | 0.64 (0.51 - 0.76) | 0.26 (0.00 - 0.48) | 0.27 (0.00 - 0.41) | 0.89 (0.86 - 0.92) |
| ABL1 | 0.51 (0.39 - 0.63) | 0.00 (0.00 - 0.13) | 0.00 (0.00 - 0.16) | 0.86 (0.86 - 0.88) |
| CEACAM1 | 0.54 (0.41 - 0.66) | 0.17 (0.04 - 0.35) | 0.20 (0.06 - 0.33) | 0.88 (0.87 - 0.91) |
| FADD | 0.55 (0.44 - 0.66) | 0.00 (0.00 - 0.13) | 0.00 (0.00 - 0.16) | 0.86 (0.86 - 0.88) |
| ADAMTS15 | 0.52 (0.39 - 0.65) | 0.13 (0.00 - 0.26) | 0.16 (0.00 - 0.27) | 0.88 (0.86 - 0.89) |
| CD27 | 0.61 (0.47 - 0.74) | 0.26 (0.09 - 0.52) | 0.27 (0.11 - 0.43) | 0.89 (0.87 - 0.93) |
| HGF | 0.51 (0.38 - 0.64) | 0.13 (0.00 - 0.30) | 0.16 (0.00 - 0.31) | 0.88 (0.86 - 0.90) |
| KLK11 | 0.59 (0.46 - 0.70) | 0.13 (0.00 - 0.30) | 0.16 (0.00 - 0.31) | 0.88 (0.86 - 0.90) |
| ANXA1 | 0.50 (0.37 - 0.62) | 0.09 (0.00 - 0.22) | 0.11 (0.00 - 0.24) | 0.87 (0.86 - 0.89) |
| FLT4 | 0.50 (0.36 - 0.62) | 0.13 (0.00 - 0.26) | 0.16 (0.00 - 0.27) | 0.88 (0.86 - 0.89) |
| GPC1 | 0.57 (0.42 - 0.71) | 0.26 (0.09 - 0.48) | 0.27 (0.11 - 0.41) | 0.89 (0.87 - 0.92) |

Table E. Stack logistic regression model coefficient estimates and respective 95% confidence interval (CI) limits and p values. See statistical analysis sub-section in Methods for the detailed description of the stacking procedure. This model was developed in the discovery set.

|  | **Coefficient Estimate** | **CI 2.5%** | **CI 97.5%** | **p value** |
| --- | --- | --- | --- | --- |
| Intercept | -1.47 | -2.34 | -0.73 | 0.00033 |
| **Diagnosis** |  |  |  |  |
| Healthy | 2.50 | 1.75 | 3.45 | 5.41×10^-9^ |
| Chronic Pancreatitis | 2.30 | 1.57 | 3.20 | 2.26×10^-8^ |
| IgG4 Disease | 2.08 | 1.26 | 3.07 | 4.98×10^-6^ |
| Irritable Bowel Syndrome | 1.92 | 1.08 | 2.86 | 1.61×10^-5^ |
| Other Biliary Duct Disease | 2.29 | 1.15 | 3.56 | 0.00016 |
| Sphincter of Oddi Dysfunction | 1.64 | 0.73 | 2.65 | 0.00063 |
| No Relevant Diagnosis | -1.73 | -3.23 | -0.39 | 0.015 |
| Other Cancer | 0.77 | 0.076 | 1.51 | 0.032 |
| Pancreatic Cyst | -1.52 | -3.02 | -0.17 | 0.035 |
| Gastritis/Reflux Disease | 0.53 | -0.44 | 1.53 | 0.28 |
| Familial Pancreatic Cancer | 0.49 | -0.41 | 1.41 | 0.29 |
| Acute Pancreatitis | 0.34 | -0.29 | 1.027 | 0.31 |
| Liver Disease | 0.38 | -0.35 | 1.14 | 0.31 |
| Isolated LFTs Derangement | -0.63 | -2.03 | 0.60 | 0.34 |
| Non-Specific Abdominal Pain | 0.301 | -0.47 | 1.05 | 0.43 |
| Gallstone Disease | 0.18 | -0.67 | 1.07 | 0.68 |

**Table F. Performance under different subsampling routines.** Oversampling corresponds to random over sampling of the minority class. Undersampling corresponds to under sampling of the majority class. SMOTE represents the Synthetic Minority Oversampling Technique (5). All subsampling is done to a ratio of 1 between majority and minority classes. Algorithm ‘Stack’ is the proposed diagnosis-based ensemble model. See Methods section in the main text for further details.

| **Algorithm** | **Subsampling** | **ROC** | **Sens at 90% Spec** | **PPV at 90% Spec** | **NPV at 90% Spec** |
| --- | --- | --- | --- | --- | --- |
| **Discovery** |  |  |  |  |  |
| Stack | Oversampling | 0.98 (0.98 - 0.99) | 0.99 (0.98 - 1.0) | 0.92 (0.91 - 0.92) | 0.99 (0.97 - 1.0) |
|  | Undersampling | 0.99 (0.98 - 1.0) | 0.99 (0.98 – 1.0) | 0.92 (0.91 – 0.92) | 0.98 (0.96- 1.0) |
|  | SMOTE | 0.99 (0.98 - 1.0) | 0.99 (0.99 - 1.0) | 0.92 (0.91 - 0.92) | 0.99 (0.98 - 1.0) |
| xgbTree | Oversampling | 0.97 (0.95-0.99) | 0.92 (0.83-1.0) | 0.40 (0.38-0.42) | 0.99 (0.99 -1.0) |
|  | Undersampling | 0.93 (0.90-0.97) | \|0.79 (0.54 - 0.96) | 0.36(0.28-0.41) | 0.98 (0.96 - 1.0) |
|  | SMOTE | 0.96 (0.94 - 0.98) | 0.96 (0.83 - 1.0) | 0.41 (0.38 -0.42) | 1.0 (0.99 - 1.0) |
| RRF | Oversampling | 0.88 (0.82 - 0.93) | 0.54 (0.33 - 0.75) | 0.28 (0.19 - 0.35) | 0.96 (0.95 - 0.98) |
|  | Undersampling | 0.88 (0.82 - 0.94) | 0.62 (0.42 - 0.83) | 0.31 (0.23 - 0.38) | 0.97 (0.96 - 0.99) |
|  | SMOTE | 0.89 (0.83 - 0.94) | 0.67 (0.29 - 0.88) | 0.32 (0.17 -0.39) | 0.97 (0.95 - 0.99) |
| RFE glm | Oversampling | 0.86 (0.79 - 0.93) | 0.71 (0.42 - 0.88) | 0.38 (0.27 - 0.43) | 0.97 (0.95 - 0.99) |
|  | Undersampling | 0.80 (0.71 - 0.89) | 0.33 (0.17 - 0.58) | 0.19 (0.11 - 0.30) | 0.95 (0.94 - 0.97) |
|  | SMOTE | 0.80 (0.70 - 0.90) | 0.65 (0.46 - 0.81) | 0.32 (0.25-0.37) | 0.97 (0.96 - 0.99) |
| **Validation** |  |  |  |  |  |
| Stack | Oversampling | 0.95 (0.91 -0.99) | 0.86 (0.68 - 1.0) | 0.54 (0.48 -0.58) | 0 98 (0.95 - 1.0) |
|  | Undersampling | 0.82 (0.71 – 0.92) | 0.55 (0.32 - 0.77) | 0.43 (0.30 - 0.52) | 0.94 (0.91 - 0.97) |
|  | SMOTE | 0.83 (0.73 - 0.93) | 0.64 (0.11 - 0.82) | 0.47 (0.36 - 0.53) | 0.95 (0.92 - 0.97) |
| xgbTree | Oversampling | 0.93 (0.89 - 0.97) | 0.65 (0.48 - 0.96) | 0.49 (0.41-0.58) | 0.95 (0.92 - 0.99) |
|  | Undersampling | 0.85 (0.78-0.91) | 0.40 (0.18 - 0.62) | 0.37 (0.21-0.47) | 0.91 (0.88 -0.94) |
|  | SMOTE | 0.96 (0.93 -0.99) | 0.87 (0.65 - 1.0) | 0.56 (0.49 - 0.59) | 0.98 (0.95 - 1.0) |
| RRF | Oversampling | 0.89 (0.83 - 0.95) | 0.61 (0.39 - 0.83) | 0.47 (0.36 - 0.54) | 0.94 (0.91 - 0.97) |
|  | Undersampling | 0.92 (0.87-0.97) | 0.74 (0 52 - 0.91) | 0.52 (0.43 -0.57) | 0.96 (0.93 - 0.99) |
|  | SMOTE | 0.88 (0.78 -0.98) | 0.74 (0.57 - 0.96) | 0.52 (0.45 -0.58) | 0.96 (0.93 - 0.99) |
| RFE glm | Oversampling | 0.83 (0.75 - 0.92) | 0.52 (0.30 - 0.70) | 0.43 (0.31-0.50) | 0.93 (0.90-0.95) |
|  | Undersampling | 0.78 (0.68-0.89) | 0 52 (0.22 - 0.74) | 0.43 (0.24-0.52) | 0.93 (0.89-0.96) |
|  | SMOTE | 0.72 (0.62-0.83) | 0.51 (0.32 - 0.68) | 0.43 (0.32-0.50) | 0.93 (0.90 -0.95) |

Table G. Values corresponding to Fig 4 (main text). Only ADEPTS samples were considered for this association study. Given that symptoms were not considered in the training of the classifiers, we concatenate ADEPTS samples in the discovery and validation sets to verify the associations of symptoms and PDAC. A univariate logistic regression model with bias correction was used for each symptom to test the association with PDAC.

| **Symptoms** | **Number of subjects** | | | | **OR (95% CI)** | **p value** |
| --- | --- | --- | --- | --- | --- | --- |
|  | Yes | | No | |  |  |
|  | Control | Case | Control | Case |  |  |
| Jaundice | 18 | 22 | 401 | 23 | 20.78 (9.98 - 44.35) | 3.22×10^-15^ |
| Weight Loss | 39 | 17 | 380 | 28 | 5.91 (2.96 - 11.62) | 1.44×10^-06^ |
| Asymptomatic | 96 | 3 | 323 | 42 | 0.28 (0.07- 0.74) | 0.0077 |
| Reflux | 38 | 0 | 381 | 45 | 0.11 (0.00 - 0.79) | 0.022 |
| Bloating | 31 | 0 | 388 | 45 | 0.14 (0.00 - 0.99) | 0.048 |
| Dyspepsia | 30 | 0 | 389 | 45 | 0.14 (0.00 - 1.03) | 0.054 |
| Abdominal Pain | 198 | 16 | 221 | 29 | 0.62 (0.33 - 1.16) | 0.14 |
| Nausea | 20 | 0 | 399 | 45 | 0.21 (0.00 - 1.60) | 0.17 |
| Vomiting | 19 | 4 | 400 | 41 | 2.23 (0.67 - 6.03) | 0.17 |
| Asymptomatic  LFT Derangement | 52 | 8 | 367 | 37 | 1.59 (0.67 - 3.39) | 0.28 |
| Anaemia | 24 | 1 | 395 | 44 | 0.54 (0.06 - 2.18) | 0.44 |
| Back Pain | 1 | 0 | 418 | 45 | 3.07 (0.02 - 58.34) | 0.54 |
| Heartburn | 9 | 0 | 410 | 45 | 0.47 (0.00 - 3.85) | 0.57 |
| Change In Bowel Habit | 58 | 7 | 361 | 38 | 1.20 (0.49 - 2.62) | 0.67 |
| Rectal Bleeding | 10 | 1 | 409 | 44 | 1.31 (0.14 - 5.80) | 0.76 |
| Dysphagia | 16 | 1 | 403 | 44 | 0.82 (0.09 - 3.42) | 0.82 |

Table H. Performance model rank summary for selected models in symptomatic patients. The probability values used to calculate the performance metrics were generated with each model developed in the training set and reported in the main text. Probability values for symptomatic patients belonging to the training set and validation set were concatenated to generate the ROC curves. Only ADEPTS samples had symptoms information. A. L. Derang.: Asymptomatic LFT Derangement. B. Pain: Back Pain. C. B. Habit: Change in Bowel Habit. W. Loss: Weight Loss. Here, only the ranks of the performances are provided. For the respective performance values see Table 2 in the main text.

|  |  | **Symptom (Yes)** | | | | |  |  |
| --- | --- | --- | --- | --- | --- | --- | --- | --- |
| **Models** | **Metric** | A.L.Derang. | A.Pain | A.B. Habit | W. Loss | Jaundice | **Geometric mean rank** | **Mean across all metrics** |
| CA19-9 | ROC | 3 | 3 | 3 | 3 | 3 | 3.00 | 2.83 |
|  | Sens90 | 3 | 3 | 2 | 3 | 3 | 2.77 |  |
|  | PPV90 | 3 | 3 | 2 | 3 | 3 | 2.77 |  |
|  | NPV90 | 3 | 3 | 2 | 3 | 3 | 2.77 |  |
| Index  signature | ROC | 1 | 1 | 1 | 1 | 1 | 1.00 | 1.16 |
|  | Sens90 | 2 | 1 | 1 | 1 | 2 | 1.32 |  |
|  | PPV90 | 2 | 1 | 1 | 1 | 1 | 1.15 |  |
|  | NPV90 | 1 | 1 | 1 | 1 | 2 | 1.15 |  |
| Reduced  signature | ROC | 2 | 2 | 2 | 2 | 2 | 2.00 | 1.79 |
|  | Sens90 | 1 | 2 | 3 | 2 | 1 | 1.64 |  |
|  | PPV90 | 1 | 2 | 3 | 2 | 2 | 1.89 |  |
|  | NPV90 | 1 | 2 | 3 | 2 | 1 | 1.64 |  |

Table I. Pairwise area under receiver operating characteristic curve comparison p-values for the selected models in Table 2 (main text). Only ADEPTS samples had symptoms information. A. L. Derang.: Asymptomatic LFT Derangement. B. Pain: Back Pain. C. B. Habit: Change in Bowel Habit. W. Loss: Weight Loss. For the respective performance values see Table 2 in the main text. Models in rows for each symptom are compared with those in the columns for the same symptom. 10000 bootstraps were constructed to test the significance of the difference in performance being lower.

| **Symptom (Yes)** |  | **Reduced signature** | **Index signature** |
| --- | --- | --- | --- |
| **A.L.Derang.** | CA19-9 | 3.89×10^-07^ | 7.70×10^-26^ |
|  | Reduced signature | - | 0.25 |
| **A. Pain** | CA19-9 | 2.19×10^-15^ | 2.83×10^-93^ |
|  | Reduced signature | - | 1.08×10^-06^ |
| **A. B. Habit** | CA19-9 | 0.0018 | 1.48×10^-28^ |
|  | Reduced signature | - | 0.020 |
| **W.Loss** | CA19-9 | 2.04×10^-09^ | 1.38×10^-14^ |
|  | Reduced signature |  | 0.018 |
| **Jaundice** | CA19-9 | 0.013 | 3.34×10^-07^ |
|  | Reduced signature | - | 0.069 |

**Table J. Quantitative ELISA assays` intra-assay coefficient of variation**.

| **Assay** | **Dilution factor** | **CV(%)** |
| --- | --- | --- |
| CA19-9(A) | 1:4 | 6.9 |
| VWF | 1:100 | 13.5 |
| THBS2 | 1:10 | 12.5 |
| PKM/PKM2 | 1:10 | 10.3 |
| IL6ST/IL6RB | 1:100 | - |

**Table K. Cancer-associated proteins measured on the Olink Oncology II panel used in this project.** The remaining biomarkers were done in-house. The protein names are listed to be consistent with those provided by Olink.

| PODXL | VEGF-A | VEGFR-2/KDR | VEGFR-3/LFT4 | RSPO3 | IL6 | IFN-gamma-R1/IFNGR1 |
| --- | --- | --- | --- | --- | --- | --- |
| CXCL17 | MK/MDK | MIC-A/B | WFDC2 | ESM-1 | MSLN | CEACAM1 |
| ITGAV | GPC1 | SYND1/SDC1 | PVRL4/NECTIN4 | TXLNA | Gal-1/GAL | LYPD3 |
| IGF1R | LYN | ABL1 | EPHA2 | CDKN1A | PPY | SCF/KITLG |
| EGF | AREG/AR | ErbB2/HER2 | ErbB3/HER3 | ErbB4/HER4 | WISP-1/CCN4 | WIF-1 |
| TRAIL/TNFSF10 | TNFSF13 | TNFRSF19 | TNFRSF6B | TLR3 | SMAD5/MAD5 | FADD |
| KLK8/hK8 | KLK11/hK11 | KLK13 | KLK14/hK14 | CPE | XPNPEP2 | CTSV |
| TFPI-2 | SCAMP3 | GZMB | GZMH | CYR61/CCN1 | ADAM8 | ADAM-TS 15 |
| FCRLB | TCL1A | CD27 | CD48 | CD70 | CD160 | CD207 |
| ANXA1 | S100A4 | S100A11 | VIM | CRNN | DLL1 | SEZ6L |
| FOLR3/FRgamma | 5’-NT/NT5E | LY9 | CA9/CAIX | FGF-BP1 | ICOSLG | FOLR1/FR-alpha |
| MIA | CEACAM5/CEA | SPARC | HGF | TGFR-2/TGFRB2 | FASLG/FasL | MetAP 2 |
| CXCL13 | ITGB5 | RET | TGF-alpha/TGFA | TNFRSF4 | GPNMB | FUR/FURIN |
| MUC16 |  |  |  |  |  |  |

**Table M. Optimal hyperparameters for xgbTree, RRF, RFE glm and the diagnosis-based ensemble.** These were found by leave one out cross validation. See Methods section in the main text for details on the underlying datasets. See Methods in the main text for further details.

| **Algorithm** | **Hyperparameters** | **Value** |
| --- | --- | --- |
| xgbTree | nrounds | 806 |
|  | max_depth | 3 |
|  | eta | 0.38 |
|  | gamma | 0.26 |
|  | subsample | 0.34 |
|  | colsample_bytree | 0.63 |
|  | min_child_weight | 1 |
| RRF | mtry | 47 |
|  | coefReg | 0.73 |
|  | coefImp | 0.0071 |
| RFE glm | Number of optimal features | 9 |
| Ensemble | Number of optimal features | 44 |

Table N. Type and number of subjects with other cancers in the ADEPTS cohort. See also Fig 1 (main text).

| **Other Cancer** | **Number of subjects** |
| --- | --- |
| Possible gallbladder cancer | 1 |
| Low grade dysplasia on ampulla of Vater (Incidental finding) | 1 |
| Hilar cholangiocarcinoma, treated Nov 2017 | 1 |
| Low anal moderately differentiated adenocarcinoma | 1 |
| Cholangiocarcinoma, primary sclerosing cholangitis | 1 |
| PNET (insulinoma) | 1 |
| Bowel cancer in 2011 | 1 |
| Prostate cancer | 1 |

**References:**

1. Stecher C, Battin C, Leitner J, Zettl M, Grabmeier-Pfistershammer K, Höller C, et al. PD-1 Blockade Promotes Emerging Checkpoint Inhibitors in Enhancing T Cell Responses to Allogeneic Dendritic Cells. Frontiers in Immunology. 2017;8.

2. Riquelme E, Zhang Y, Zhang L, Montiel M, Zoltan M, Dong W, et al. Tumor Microbiome Diversity and Composition Influence Pancreatic Cancer Outcomes. Cell. 2019;178(4):795-806 e12.

3. Peng H, James CA, Cullinan DR, Hogg GD, Mudd JL, Zuo C, et al. Neoadjuvant FOLFIRINOX Therapy Is Associated with Increased Effector T Cells and Reduced Suppressor Cells in Patients with Pancreatic Cancer. Clinical Cancer Research. 2021;27(24):6761-71.

4. Wang C, Li X, Zhang L, Chen Y, Dong R, Zhang J, et al. miR-194-5p down-regulates tumor cell PD-L1 expression and promotes anti-tumor immunity in pancreatic cancer. Int Immunopharmacol. 2021;97:107822.

5. Blagus R, Lusa L. SMOTE for high-dimensional class-imbalanced data. BMC Bioinformatics. 2013;14:106.
